# RecN and RecA orchestrate an ordered DNA supercompaction response following ciprofloxacin-induced DNA damage in *Escherichia coli*

**DOI:** 10.1093/nar/gkaf437

**Published:** 2025-05-28

**Authors:** Krister Vikedal, Synnøve Brandt Ræder, Ida Mathilde Marstein Riisnæs, Magnar Bjørås, James Alexander Booth, Kirsten Skarstad, Emily Helgesen

**Affiliations:** Department of Microbiology, University of Oslo and Oslo University Hospital, Rikshospitalet, 0373 Oslo, Norway; Department of Microbiology, University of Oslo and Oslo University Hospital, Rikshospitalet, 0373 Oslo, Norway; Department of Microbiology, University of Oslo and Oslo University Hospital, Rikshospitalet, 0373 Oslo, Norway; Department of Microbiology, University of Oslo and Oslo University Hospital, Rikshospitalet, 0373 Oslo, Norway; Department of Clinical and Molecular Medicine, Norwegian University of Science and Technology, 7030 Trondheim, Norway; Department of Microbiology, University of Oslo and Oslo University Hospital, Rikshospitalet, 0373 Oslo, Norway; Department of Clinical and Molecular Medicine, Norwegian University of Science and Technology, 7030 Trondheim, Norway; Department of Microbiology, University of Oslo and Oslo University Hospital, Rikshospitalet, 0373 Oslo, Norway; Department of Microbiology, University of Oslo and Oslo University Hospital, Rikshospitalet, 0373 Oslo, Norway; Department of Clinical and Molecular Medicine, Norwegian University of Science and Technology, 7030 Trondheim, Norway

## Abstract

Fluoroquinolones induce double-strand breaks in bacterial DNA, triggering the SOS response, a major DNA damage response that ensures the expression of repair proteins but also promotes the emergence and spread of antibiotic resistance. Fluoroquinolone resistance, particularly in *Escherichia coli*, is a growing global health concern. Understanding bacterial responses to these antibiotics is critical for developing preventive strategies and novel treatments to combat resistance development. This study investigates DNA morphology in *E. coli* following exposure to ciprofloxacin (CIP), a fluoroquinolone antibiotic. We show that CIP induces a stepwise DNA reorganization, culminating in a highly dense nucleoid structure at midcell—a process we term DNA supercompaction. This phenomenon occurred also with other genotoxic agents. Live-cell imaging revealed that RecN, a structural maintenance of chromosomes (SMC)-like protein, is required for DNA supercompaction, and that RecN’s dynamics and activity in this response depend on RecA. Additionally, RecN and RecA frequently colocalized at nucleoid-associated positions. We suggest that RecN and RecA play active roles in DNA supercompaction following severe DNA damage, that their interplay is part of a prompt universal survival response to DNA double-strand breaks in *E. coli*, and that the extent of the compaction response depends on the DNA damage severity.

## Graphical Abstract

**Graphical Abstract:**
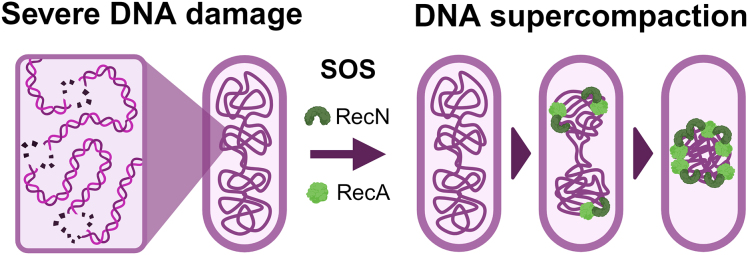

## Introduction

Resistance to fluoroquinolone-class antibiotics poses a growing global health concern [1]. In 2019, fluoroquinolone resistance was responsible for approximately a quarter of worldwide deaths attributable to antimicrobial resistance, with most of these deaths linked to fluoroquinolone-resistant *Escherichia coli* [1]. These antibiotics, such as ciprofloxacin (CIP), target and inhibit DNA gyrase and topoisomerase IV, enzymes critical for maintenance of DNA supercoiling and important for DNA replication and repair. Inhibition of these enzymes by CIP leads to DNA double-strand breaks (DSBs), ultimately resulting in cell death [2, 3].

*E. coli* respond to DSBs through highly efficient, conserved mechanisms to preserve chromosomal integrity and ensure survival [4]. A major repair pathway is homologous recombination, where intact double-stranded DNA (dsDNA) serves as a template to repair broken DNA [5, 6]. RecBCD and RecA enzymes play pivotal roles in this process [7]. RecBCD processes DSB ends to create single-stranded DNA (ssDNA) [8, 9]. Upon recognition of the Chi sequence by RecBCD, RecA is loaded onto these ssDNA regions, forming RecA–ssDNA filaments that ultimately facilitate the search for and invasion of homologous dsDNA to mediate genetic exchange and repair [6, 10, 11].

Besides its central role in homologous recombination, RecA is also critical for the induction of the SOS response, the major DNA damage response. RecA–ssDNA filaments promote the autocatalytic degradation of the SOS response repressor LexA, triggering the expression of genes needed for DSB repair and survival [12–14]. The SOS response not only governs DNA repair mechanisms but also contributes to the emergence and spread of antibiotic resistance [15, 16]. Therefore, understanding the facets of the SOS response is critical for developing strategies to manage increasing antibiotic resistance.

DNA damage caused by genotoxic agents such as mitomycin C (MMC), nalidixic acid, or UV irradiation induces significant morphological changes in the bacterial nucleoid [17–21]. These agents have been associated with varying DNA compaction phenotypes and kinetics. For instance, nalidixic acid exposure has been observed to result in a dense, persistent midcell nucleoid [17], whereas UV irradiation can lead to transient DNA compaction at both quarter and midcell positions followed by prolonged decompaction [18]. Similarly, MMC exposure has been reported to trigger transient compaction followed by decompaction [19]. Notably, RecA is necessary for DNA compaction induced by UV and MMC but has been reported not to be required for the initial stages of compaction induced by nalidixic acid [17–19]. Another critical player in the DNA damage response is the structural maintenance of chromosomes (SMC)-like protein RecN, which influences both DNA compaction and repair after exposure to genotoxic agents [18, 20]. It remains to be determined whether the various DNA compaction phenomena aid DNA repair or are instead consequences of macromolecular crowding and other intracellular changes related to unknown survival mechanisms.

RecN, a highly conserved protein, is one of the most abundantly produced proteins during the SOS response [13, 22, 23]. The *recN* gene is rapidly expressed in response to DNA damage [13, 24] but is also rapidly digested by the ClpXP protease system to ensure high levels only upon DNA damage [25, 26]. RecN has been suggested to play a crucial role in aiding DSB repair, and mutants lacking RecN exhibit heightened sensitivity to ionizing radiation, MMC, and I-SceI induced DSBs [27–29].

The exact mechanism by which RecN aids DSB repair is not fully understood. In vitro studies have revealed that RecN binds preferentially to RecA-bound ssDNA at DSBs [30], enabling it to bridge a dsDNA molecule via ATP-dependent topological entrapment [23, 30–32]. *In vivo*, RecN has been shown to promote contacts between newly replicated homologous DNA strands after MMC exposure [19]. Green fluorescent protein (GFP)-tagged RecN localizes to the nucleoid following DNA damage [26], and colocalization with RecA at nucleoid gaps upon MMC exposure has been observed [33]. Additionally, RecA is necessary for RecN nucleoid localization, and RecA repair activities appear to depend on RecN [33, 34]. Importantly, cells lacking RecN are unable to perform DNA compaction effectively after exposure to various genotoxic agents [18, 20].

In this study, we investigate the change in DNA morphology following CIP exposure in *E. coli*. Our findings reveal that CIP exposure leads to a stepwise reorganization of DNA towards midcell—a process we term DNA supercompaction. This process was also induced by other fluoroquinolones and MMC, indicating that it is part of a universal response to genotoxic agents. Notably, we find that the process requires both RecN and RecA, in what appears to be an active interplay. We suggest that DNA supercompaction is part of a prompt universal survival response to severe DNA damage.

## Materials and methods

### Strain construction

Strains and plasmids used in this study are listed in Tables 1 and 2, respectively. Most strains are derived from the *E. coli* K-12 strain BW25113 [35], which serves as the background strain of the Keio collection [36]. To examine the effect of *recN* deletion, we utilized the Keio collection strain JW5416. Standard P1 transduction procedures [37] were used to knock out *recA* and introduce *hupA100*::*mCherry* (generous gift from Steven Sandler [38, 39]) for visualization of DNA in the relevant backgrounds (see Table 1). FLP recombinase (pCP20) [40] was used to remove the *kan*-cassette when both donor and recipient strains carried kanamycin resistance.

**Table 1.** *E. coli* strains used in this study. Relevant antibiotic resistances are shown in brackets. P1 transduction is shown as: P1 Donor x Recipient

Strain	Relevant genotype	Source
BW25113	Wild-type	[35]; CGSC7636
SS6282	MG1655 *hupA100*::*mCherry*::*kan ΔattB*::*psulA-gfp* [Kan^R^]	[39]; S. Sandler
KV21	BW25113 *hupA100*::*mCherry*	This work; P1 SS6282 x BW25113, kan removed with pCP20
JW5416	BW25113 *ΔrecN*::*kan* [Kan^R^]	[36]; Keio collection
MR09	BW25113 *ΔrecN*	This work; kan removed from JW5416 with pCP20
MR16	BW25113 *ΔrecN hupA100*::*mCherry*::*kan* [Kan^R^]	This work; P1 SS6282 x MR09
KV60	KV21 + pSG101 (pSOS) [Amp^R^]	This work
KV61	KV21 + pTF271 (pARA) [Amp^R^, Cam^R^]	This work
KV62	MR16 + pSG101 (pSOS) [Kan^R^, Amp^R^]	This work
KV63	MR16 + pTF271 (pARA) [Kan^R^, Amp^R^, Cam^R^]	This work
JW2669	BW25113 *ΔrecA*::*kan* [Kan^R^]	[36]; Keio collection
KV66	KV61 *ΔrecA*::*kan* + pSG101 (pSOS) [Kan^R^, Amp^R^]	This work; P1 JW2669 x KV60
KV67	KV61 *ΔrecA*::*kan* + pTF271 (pARA) [Kan^R^, Amp^R^, Cam^R^]	This work; P1 JW2669 x KV61
KV68	BW25113 *ΔrecN hupA100*::*mCherry*	This work; kan removed from MR16 with pCP20
KV69	BW25113 *ΔrecN ΔrecA::kan hupA100*::*mCherry* [Kan^R^]	This work; P1 JW2669 x KV68
KV70	KV69 + pSG101 (pSOS) [Kan^R^, Amp^R^]	This work
KV71	KV69 + pTF271 (pARA) [Kan^R^, Amp^R^, Cam^R^]	This work
KV75	BW25113 *recA*::*mCherry* [Cam^R^]	This work; PMGR in BW25113 with pDL5196
KV76	JW5416 *recA*::*mCherry* [Kan^R^, Cam^R^]	This work; PMGR in JW5416 with pDL5196
KV77	KV75 + pSG101 (pSOS) [Cam^R^, Amp^R^]	This work
KV78	KV76 + pSG101 (pSOS) [Kan^R^, Cam^R^, Amp^R^]	This work
KV79	KV75 + pTF271 (pARA) [Cam^R^, Amp^R^]	This work
KV80	KV76 + pTF271 (pARA) [Kan^R^, Cam^R^, Amp^R^]	This work
KP84^a^	*lexA41* (*lexA3*) [Tet^R^]	[44]; Lab collection
KV90^a^	*lexA41* (*lexA3*) *hupA100*::*mCherry*::*kan* [Kan^R^, Tet^R^]	This work; P1 SS6282 x KP84
KV91^a^	*lexA41* (*lexA3*) *hupA100*::*mCherry* [Tet^R^]	This work; kan removed from KV90 with pCP20
KV92^a^	*lexA41* (*lexA3*) *ΔrecA*::*kan hupA100*::*mCherry* [Kan^R^, Tet^R^]	This work; P1 JW2669 x KV91
BTH101	*cya-99* [Str^R^]	Euromedex (Cat. no. EUK001)

^a^The *lexA41* (*lexA3*) mutations correspond to the point mutations *lexA*_G85D, A132T_, as determined from sequencing data of the KP84 strain.

PMGR = Plasmid-mediated gene replacement; Kan^R^ = Kanamycin resistance; Cam^R^ = Chloramphenicol resistance; Amp^R^ = Ampicillin resistance; Tet^R^ = Tetracycline resistance; Str^R^ = Streptomycin resistance.

**Table 2.** Plasmids used for main experiments in this study

Plasmid	Description	Source
pSG101 (pSOS)	pSCH19 derivative with N-terminal enhanced GFP-tagged RecN controlled by its original SOS promoter [Amp^R^]	[26]; T. Hishida
pTF271 (pARA)	pTF200 derivative with N-terminal enhanced GFP-tagged RecN controlled by an arabinose-inducible promoter [Amp^R^, Cam^R^]	[26]; T. Hishida
pDL5196	pTOF_24_ derivative for endogenous integration of *recA*::*mCherry* between *recA* and *recX* genes [Cam^R^, *repA*^ts^, Suc^S^]	[41]; D. Leach

Cam^R^ = Chloramphenicol resistance; Amp^R^ = Ampicillin resistance; *repA*^ts^ = Temperature-sensitive origin of replication (replicates at 30°C); Suc^S^ = Sucrose sensitive (5% wt/vol).

For studying the localization and dynamics of RecN, we employed plasmids encoding GFP–RecN (generous gifts from Takashi Hishida [26]), regulated either by the native SOS promoter (pSOS) or an arabinose-inducible promoter (pARA). The plasmids were purified using a Miniprep kit (Qiagen, cat. no. 27104) and introduced into relevant backgrounds using electroporation transformation (see details below).

To visualize RecA, *recA-mCherry* was used, chromosomally expressed in tandem with the endogenous *recA* gene. This construct was introduced by plasmid-mediated gene replacement (PMGR) (see details below), following electroporation transformation of the pDL5196 plasmid (generous gift from David Leach [41]) into relevant backgrounds.

### Growth conditions

Strains were streaked directly from frozen stocks onto LB-agar plates and incubated overnight. The next day, 5–10 colonies of each strain were picked and incubated in LB medium overnight with shaking. On the day of the experiment, the overnight cultures (ONCs) were diluted 1:200 into fresh LB medium and cultured at 37°C to exponential phase (OD_600_ ∼0.4) before treatment or fixation unless otherwise specified. For strains carrying the pARA plasmid, arabinose (0.05% final concentration) was added when OD_600_ reached 0.15, and the cultures were incubated for another 60 min before CIP treatment. A dose of 10 μg/ml CIP was used in this study, except in specified cases where 500 ng/ml or the minimum inhibitory concentration (MIC) of 20 ng/ml was used. Cells were exposed to a similar variation of doses relative to MIC when using the genotoxic antibiotics norfloxacin (100 ng/ml and 10 μg/ml), ofloxacin (200 ng/ml and 10 μg/ml), nalidixic acid (4 and 100 μg/ml), and MMC (1, 10, and 50 μg/ml). Other antibiotics were used as indicated in Tables 1 and 2 with the following concentrations: 30 μg/ml kanamycin, 100 μg/ml ampicillin, 20 μg/ml chloramphenicol, 5 μg/ml tetracycline, and 100 μg/ml streptomycin.

### Electroporation transformation

Electroporation was used to transform plasmids into recipient strains. The recipient strains were first cultured to exponential phase and then rendered electrocompetent through two rounds of washing and resuspension in MQ water with 10% glycerol while kept on ice. For electroporation, 50 μl of electrocompetent cells and 50–100 ng of purified plasmid were added to an electroporation cuvette with a 1 mm gap (Thermo Fisher, P41050), and an exponential pulse of 1.35 kV, 600 Ω, and 10 μF was applied using a Gene Pulser II (Bio-Rad).

Immediately after electroporation, the cells were suspended in 700 μl of SOC medium and incubated at 37°C with shaking for 1–2 h for recovery. Then, 100 μl of the recovered cell culture was plated on LB-agar with appropriate antibiotics and incubated at 37°C overnight. The next day, single transformant colonies were picked and grown to saturation before preparation of frozen stocks.

### Plasmid-mediated gene replacement

A protocol from David Leach’s lab [41], based on work by Link *et al.* [42] and Merlin *et al.* [43], was used for integration of the *recA-mCherry* construct between the chromosomal *recA* and *recX* genes using the pDL5196 plasmid. This plasmid carries *recA-mCherry*, chloramphenicol resistance (Cam^R^), a temperature-sensitive origin of replication (*repA*^ts^), and sucrose sensitivity (*sacB*) (see Table 2).

The pDL5196 plasmid was introduced into the strain of interest via electroporation transformation, recovering cells at 30°C before they were plated on LB plates containing chloramphenicol for selection. To eliminate the pDL5196 plasmid and select for cells that had integrated *recA*-*mCherry* through recombination, colonies were restreaked onto chloramphenicol plates and incubated at 42°C overnight. This selection process was repeated the next day. To confirm plasmid loss, individual colonies were cultured in LB without chloramphenicol overnight and screened on LB-sucrose plates. Finally, the presence of mCherry fluorescence was verified by fluorescence microscopy.

### Live-cell imaging with microfluidic setup

To examine the cells’ immediate response to CIP exposure, we employed a microfluidic setup to image cells using a Zeiss Axio Observer Z1 widefield inverted microscope with a 63x oil objective (Zeiss Plan Apochromat 1.4 NA, DIC), a Colibri 7 LED light source, a Hamamatsu ORCA-Flash4.0 V3 digital CMOS camera, as well as a heated incubation chamber and mounting frame, both maintained at 37°C.

Cells were cultured to exponential phase and immobilized on a chitosan-coated coverslip (Chitozen, Idylle Labs, TMI-CHI-7525) within microfluidic channels (Ibidi, 80 608) following Idylle Labs’ standard protocol. Precleaned microfluidic tubes were connected to the inlet and outlet of the microfluidic channel to enable flow of growth medium. LB$\frac{1}{2}$ medium (LB diluted 1:1 with Milli-Q water) was used to reduce background noise during imaging, and care was taken to not introduce air bubbles during setup. A peristaltic pump (Ismatec, ISM930C) was used to apply medium flow through the channel at 2 ml/min for 5 min to clear nonattached cells.

Cells in the microfluidic channels were imaged at 2-min intervals and allowed to grow unchallenged for 30 min to verify normal cell growth and division. Next, medium with CIP (10 μg/ml) was introduced by applying flow for 10 min to ensure complete medium exchange. To account for focus drift, Z-stacks of seven slices at 0.50-μm intervals with manually adjusted midpoints were captured. Transmitted light (TL) and mCherry fluorescence channels were imaged using 2 × 2 binning. The mCherry was excited at 540–570 nm and emissions collected at 570–640 nm. Because of signs of bleaching or photodamage, we excluded frames after 60 min of timelapse imaging from the presented figures.

### Live-cell spinning disk microscopy

To explore details of DNA supercompaction and the interplay between RecN and RecA, we employed spinning disk microscopy with improved focus stability and reduced photodamage effects. The Nikon Eclipse Ti2-E inverted microscope was equipped with a ×60 oil objective (Nikon Plan Apochromat λD 60 × 1.42 NA, DIC), a CrestOptics X-Light V3 spinning disk confocal module (50:400 μm spinning disk), a Lumencor Celeste multi-line laser, two Teledyne Photometrics Kinetix sCMOS cameras, as well as a stage-top incubator chamber and an objective heater, both maintained at 37°C. The microscope’s Perfect Focus System (automatic focus), guided by near-infrared light, ensured that cells remained consistently in focus without requiring the use of Z-stacks.

Cells were cultured to exponential phase, as previously described. Immediately after addition of CIP, cultures were gently shaken for 1 min before 10 μl was transferred to an LB$\frac{1}{2}$-agar pad (1% agarose in LB$\frac{1}{2}$ medium) pre-made on a microscope glass slide within a Gene Frame (Thermo Scientific, no. AB0576). Once dried, the agar pad was sealed with a cover slip (#1.5 thickness). Imaging commenced 8–12 min after CIP exposure, with acquisition of one or three locations at either 10-s, 15-s, or 2-min intervals, as specified in each case.

Three channels were used for imaging: a TL channel for cell outlines and fluorescence channels for GFP and mCherry. GFP was excited at 477 nm and its emissions collected at 501–521 nm, whereas mCherry was excited at 546 nm and its emissions collected at 580–610 nm. We did not observe bleaching or photodamage throughout our timelapse experiments.

#### Imaging of strains with temperature-regulated SOS induction

To evaluate the role of RecA and SOS induction in DNA supercompaction, the strains KV90 and KV92 were used, featuring temperature-regulated SOS induction [44]. Cultures were grown at 30 or 37°C to modulate SOS induction levels, with the microscope incubation chamber and objective heater set to match these temperatures during imaging. The experimental setup was otherwise consistent with the method described above.

### Microscopy of DNA in fixed cells

The DNA compaction phenotype of strains without chromosomally expressed HU-mCherry was investigated before and after CIP exposure using conventional DNA staining with Hoechst 33258. Cells were grown to exponential phase in LB and fixed with ethanol (50% final concentration), before storage at 4°C until further use. Prior to imaging, samples were stained with 5 μg/ml Hoechst 33 258 in PBS for 10 min, washed with PBS, and up-concentrated 2–4 times.

KV75, KV76, KV77, KV78, KV79, and KV80 strains, all containing the *recA-mCherry* construct, were cultured at 37°C, with samples fixed prior to treatment and after 15 and 30 min of CIP exposure. After Hoechst staining, 10 μl of sample was added to an agar pad on a glass slide pre-made within a Gene Frame (Thermo Scientific, no. AB0576), sealed with a cover slip (#1.5 thickness) once dried.

We used a Leica DM6000 B microscope with a ×100 oil objective (Leica HCX Plan Apochromat 10× 1.40 NA, PH3 CS), a Leica EL6000 metal-halide light source, and a Hamamatsu C9100-14 EM-CCD camera. Three locations across the agar pad were imaged to include many cells in the analysis. Each image included a phase-contrast channel for cell outlines, and a fluorescence channel for Hoechst 33 258 with excitation at 334–406 nm and emission collection at 407–497 nm.

### Image processing and analysis

To process and analyze images, we used the open-source software Fiji (ImageJ) [45] and its plugins Coli-Inspector [46] and MicrobeJ [47]. Coli-Inspector was used to analyze images from the live-cell microfluidic setup. MicrobeJ was used to analyze all remaining images. All scripts and templates used for processing and analysis in this study are available in a Zenodo repository (see Data Availability).

All images were preprocessed to remove background noise, with methods depending on the microscopy setup used. For images from the microfluidic setup, only the best-focused slice from the Z-stack was selected for each time point. For images from spinning disk microscopy, flat-field correction was applied to all image channels, in addition to a Gaussian blur (1–2 pixels radius) for the TL channel, and a background subtraction with 1-μm sliding paraboloid for fluorescence channels. For the remaining imaging techniques, noise was removed from the TL channel by subtracting a 2-μm median-filtered version and from the fluorescence channels by a 1-μm sliding paraboloid background subtraction.

We customized the script of the ObjectJ-based Coli-Inspector plugin (version 03f) [46] to allow grouping of cells by image number, and thus by time frame (see Data Availability). Cells were first segmented using the default method with adjusted limitations on cell area, cell width, and circularity. We measured average symmetrical fluorescence intensity profiles along cells’ long axes for each frame and transferred these measurements to GraphPad Prism (version 10.2.0). The outer bounds of the average fluorescence peaks at 80% of their maximum intensity was determined for each time point, so that the distances between the outer bounds could be plotted to illustrate the distribution of DNA along the cells’ long axes.

For kymograph creation and DNA distribution analysis, MicrobeJ (main version 5.13p (1)) was used [47]. Cells were segmented using the “Medial Axis” mode of detection with limitations on cell area, length, width, curvature, sinuosity, and angularity. Cells that could not be tracked for at least 10 consecutive frames were excluded. An additional step was applied to detect division sites and segment cells accordingly. Single-cell kymographs were created with the kymograph plotting tool in the MicrobeJ results interface from individually tracked and analyzed cells. Kymograph heat maps were generated from multiple analyzed cells with the ShapePlot tool. For DNA distribution analysis, we measured average fluorescence intensity profiles along cells’ long axes for each frame and transferred these measurements to GraphPad Prism (version 10.2.0) for plotting.

A beta-version of MicrobeJ (beta-version 5.13p (13)) [47] was used to track and analyze GFP–RecN and RecA-mCherry foci because of issues with particle tracking coordinates in the current main version. The cell segmentation method was the same as in the current main version. Fluorescent foci of GFP–RecN, HU-mCherry, or RecA-mCherry were detected inside segmented bacteria. To quantify RecN colocalization with DNA, GFP–RecN foci inside the regions of already detected HU-mCherry foci were identified without any margin. The same approach was used to identify RecA foci colocalized with RecN, but with a 1-pixel margin. For tracking of distinct RecN foci over time, an absolute coordinate system was used. Trajectories with lifespans below five frames were discarded. The relative midcell distances of foci were calculated by dividing their absolute distance to the parent bacteria midcell by the length of the parent bacteria. All results from detected and tracked foci were transferred to GraphPad Prism (version 10.2.0) for plotting.

#### Classification of DNA compaction phenotypes

DNA compaction phenotypes of cells in live-cell images were classified using the beta-version of MicrobeJ (5.13p (13)). Cells were segmented as described above, with both focal point maxima and foci detected independently for HU-mCherry fluorescence. Focal point maxima are specific to single pixels, whereas foci are defined across an area. Based on the number of HU-mCherry focal point maxima and foci, DNA compaction phenotypes were classified as: (i) multifocal distribution, more than two focal point maxima; (ii) quarter-position compaction, two focal point maxima and more than one focus; (iii) midcell compaction, exactly one focal point maximum; and (iv) periseptal compaction, two focal point maxima and only one focus. Cells that did not fit into any of these classes were excluded from analyses. The cell count for each phenotype was transferred to GraphPad Prism (version 10.4.1) and normalized to the total cell count for each replicate. To quantify the progression rates of DNA supercompaction, we determined the interpolated timing when 10% of cells were classified with either midcell or periseptal compaction.

### Western blotting to compare RecN expression levels

ONCs were diluted 1:100 in 100 ml of LB cultures and grown to exponential phase. For strains carrying pSOS (KV60 and KV62) and the control strains (KV21 and KV68), the cultures were grown until OD_600_ reached 0.4 before half the cultures were treated with CIP (10 μg/ml) for 20 min. For strains carrying pARA (KV61 and KV63), arabinose (0.05% final concentration) was added when the OD_600_ reached 0.2, and the cultures were incubated for an additional 60 min. CIP (10 μg/ml) was added to one half of the pARA cultures for the last 20 min of incubation. All cultures, including unchallenged halves, were pelleted 20 min after CIP addition. The pellets were washed once with PBS, snap-frozen with liquid nitrogen, and resuspended in 0.5 ml of lysis buffer (50 mM Tris, pH 7.5, 150 mM NaCl, 1 mg/ml lysozyme, and 1 mM phenylmethylsulfonyl fluoride (PMSF)) while kept on ice. Samples were sonicated for 25 cycles (30 s on, 30 s off) using Bioruptor Pico (Diagenode). After sonication, samples were centrifuged at 20 000 × *g* for 20 min, and the supernatant with protein extract was retained.

Protein extracts (40 μg) from each sample was run on 4%–15% Mini-PROTEAN TGX Precast Gels (Bio-Rad) using NuPAGE LDS Sample Buffer (final 1×) and 1 mM dithiothreitol (DTT). SeeBlue Plus2 Pre-stained Protein Standard (Invitrogen) and purified his-tagged RecN protein were used as markers. The proteins were transferred to a nitrocellulose membrane (0.2 μm) using Trans-Blot Turbo Mini Transfer Packs (Bio-Rad) at 25 V, 1.35 A for 10 min in a Trans-Blot Turbo Transfer System (Bio-Rad). Afterwards, the membranes were blocked in PBS-Tween with 5% skim milk (Merck) for 45 min, followed by overnight blocking in cell extract made from the KV68 (*ΔrecN*) strain (see details below). After blocking, the membranes were incubated 2 h with primary anti-RecN antibody (rabbit anti-RecN polyclonal antibody, MyBiosource, cat. no. MBS7162137) diluted 1:200 in PBS-Tween with 5% skim milk, and then 45 min with secondary goat anti-rabbit IgG (H + L) HRP-conjugated antibody (MyBiosource, cat. no. MBS705310) diluted 1:10 000 in PBS-Tween with 5% skim milk. Finally, the membranes were incubated with SuperSignal West Pico PLUS Chemiluminescent Substrate (Thermo Scientific, cat. no. 34 577) for chemiluminescent imaging. The western blot membranes were imaged using a ChemiDoc MP Imaging System with Image Lab software (Bio-Rad).

RecN levels from western blot images were quantified using Fiji (ImageJ) [45]. The gel lane selection tool was used to select regions encompassing both the GFP–RecN (98 kDa) and RecN (61 kDa) proteins for each sample lane. The lane profiles were plotted and the measured signal from each lane was normalized to the wild-type strain after subtracting the background signal from the negative control. The final results were plotted using GraphPad Prism version 10.2.0.

#### Cell extracts for blocking of western blot membranes

To make cell extracts from *ΔrecN* cells (KV68) for blocking of unspecific binding by the anti-recN antibody, ONCs were diluted 1:100 in 1 l LB and grown to OD_600_ of 0.5 before addition of 10 μg/ml CIP. The cultures were pelleted after 20 min of CIP exposure, washed with PBS, and snap-frozen. Pellets were lysed by sonication in 10 ml of PBS with 1 mg/ml lysozyme at amplitude 60 for 30 s in three cycles using a Vibracell VC601 sonicator with a 13 mm probe (Sonics & Materials). The lysed pellets were centrifuged at 20 000 × *g* for 20 min, and the resulting supernatants were collected. Subsequently, the supernatants were incubated at 4°C for 2 h, after addition of 3 ml of PBS-Tween with 10 mM EDTA and 1:200 anti-recN antibody (rabbit anti-RecN polyclonal antibody, MyBiosource, cat. no. MBS7162137). Finally, this solution was centrifuged at 4000 × *g* for 15 min before the resulting supernatant was added to membranes as the cell extract for blocking.

### Survival assays

#### Spot assay

To examine if the presence of GFP–RecN, or total elevated RecN levels affected cell survival, we performed a spot survival assay. Cells were cultured at 37°C to an OD_600_ of ∼0.1. The cultures were then split into two batches: one batch was incubated with arabinose (0.05% final concentration) for an additional 60 min, while the other batch was incubated until its OD_600_ reached 0.4 without arabinose. Samples from each batch, before and after 20 min of CIP exposure, were serially diluted 10-fold with LB medium (10^0^–10^−9^) in 96-well plates. Samples exposed to CIP were washed once with LB before serial dilution. From each dilution, 10 μl was spotted onto LB-agar plates with or without 0.05% arabinose and incubated overnight at 37°C. Colony growth was assessed the next day and the plates imaged using a GelDoc Go imaging system (Bio-Rad).

#### Time-dependent survival assay

A time-dependent survival assay was used to compare the survival of wild-type (BW25113) and *ΔrecN* (JW5416) strains after different CIP exposure times. ONCs of each strain were diluted 1:100 and cultured to OD_600_ ∼0.3. Each culture was split into three parts in a 96-well plate: one part remained unchallenged, one was treated with the experimental dose of CIP (10 μg/ml), and one was treated with the MIC dose of CIP (20 ng/ml). A sample of 5 μl was collected from each part prior to treatment and at multiple time points from 1 to 120 min after CIP exposure. The unchallenged samples served as references. All samples were serially diluted 10-fold in 96-well plates, spotted onto LB-agar plates (5 μl), and incubated overnight at 37°C. The number of viable colonies were counted the following day to determine colony-forming units (CFU) per ml. Relative survival was calculated as the CFU/mL ratio between CIP-treated and unchallenged samples from three biological replicates.

#### Bacterial two-hybrid assay

A bacterial two-hybrid assay kit (Euromedex, cat. no. EUK001) was used to investigate direct interaction between RecN and RecA [48, 49]. The assay is based on reconstitution of two fragments (T18 and T25) into adenylate cyclase (CyaA) in a *ΔcyaA* strain (BTH101), which will lead to production and activity of β-galactosidase upon direct interaction of the assayed proteins. The *recN* and *recA* genes were cloned into pK(N)T25 and pUT18(C) vectors (GenScript) to create N- and C-terminal fusions with T25 and T18 fragments (Supplementary Table S1).

The assay was based on the kit protocol, as well as work by Mehla *et al.* [49] and Battesti and Bouveret [48]. In short, BTH101 cells were co-transformed with combinations of pUT18(C) and pK(N)T25 derivatives. Transformants were plated on LB-agar with appropriate antibiotics and incubated at 30°C for 3 days, after which ONCs were inoculated in LB with 0.5 μM isopropyl β-D-1-thiogalactopyranoside (IPTG). The next day, ONCs were diluted 1:100 into fresh LB with 0.5 μM IPTG and grown to OD_600_ ∼0.5. Cells were then pelleted, resuspended in PM2 buffer (70 mM Na_2_HPO_4_, 30 mM NaH_2_PO_4_, 1 mM MgSO_4_, and 0.2 mM MnSO_4_) containing β-mercaptoethanol (100 mM), and permeabilized with toluene (10%), and sodium dodecyl sulfate (SDS) (0.01%) at 37°C for 45 min. Afterwards, permeabilized cells were pelleted, and the supernatant transferred to 96-well polypropylene plates in three technical replicates. The supernatants were mixed with ONPG (*o*-nitrophenyl-β-D-galactoside) (0.67 mg/ml final concentration) and incubated at 30°C to produce yellow-colored *o*-nitrophenol in reaction to β-galactosidase. After 30–40 min, the reaction was stopped with sodium carbonate (200 mM), and β-galactosidase activity (*o*-nitrophenol) was measured through absorption at 405 nm (OD_405_) using a Victor Nivo multimode plate reader (PerkinElmer). The results were normalized to measurements from the positive (pKT25-zip + pUT18C-zip) control.

### Statistical analysis

Single representative replicates have been presented in figures detailing DNA distribution and RecN or RecA dynamics to accurately represent the kinetics in the population which may vary slightly between replicates. The remaining results are presented as mean ± error from biological replicates. The differences in cell percentage belonging to each of the four DNA compaction phenotypes between baseline unchallenged wild-type and *ΔrecN* cells were analyzed using an unpaired Welch *t*-test with Holm–Šidák correction. The differences in relative midcell distance between RecA foci colocalizing with RecN and noncolocalizing RecA foci were presented as 95% confidence intervals (CIs) and analyzed using one sample *t*-tests with a hypothetical value of 0 in GraphPad Prism (version 10.2.0). We considered two-tailed *P*-values <0.05 as significant and indicated this with asterisks: ns, nonsignificant; *P ≤ 0.05, **P ≤ 0.01, ***P ≤ 0.001, ****P ≤ 0.0001.

## Results

### Ciprofloxacin exposure leads to DNA supercompaction through a stepwise reorganization

Exposure to various genotoxic agents is known to induce chromosomal reorganization in bacteria [17–21, 50, 51]. Given the widespread resistance to CIP, we investigated if and how CIP induces chromosomal reorganization in *E. coli*. For this purpose, we used live-cell imaging to monitor DNA morphology in exponentially growing cells containing HU-mCherry for DNA visualization (KV21, hereafter referred to as wild-type).

**Figure 1.**
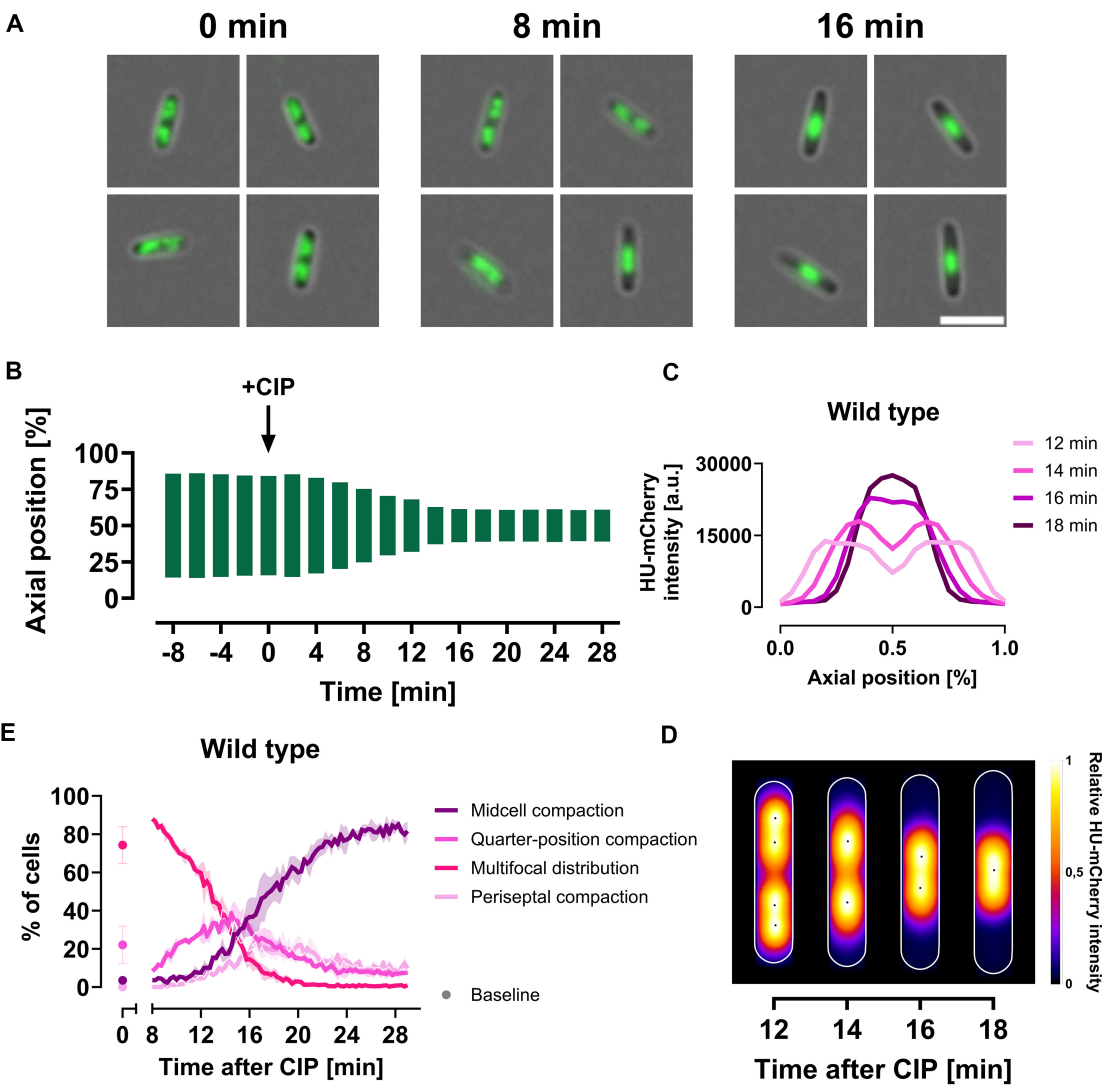
DNA supercompaction in wild-type *E. coli* cells (KV21) after CIP exposure. All cells were grown in LB at 37°C and imaged at 2-min intervals using live-cell imaging. (**A** and **B**) Cells were immobilized in microfluidic channel slides and imaged using widefield microscopy. (A) Representative images of wild-type cells with HU-mCherry fluorescence (green) at 0, 8, and 16 min after CIP exposure, showing DNA distribution; scale bar: 5 μm. (B) Analysis of DNA distribution along the cells’ long axis before and after CIP, quantified by measuring the distance between the outer bounds of symmetrical fluorescence peaks at 80% of maximum averaged intensity for each time point. Results are averaged from 34 to 141 cells from a single representative biological replicate (see “Materials and methods” section for detailed explanation). (**C** and **D**) Cells were immobilized on agar pads and imaged using spinning disk microscopy. Results shown are from images captured 12, 14, 16, and 18 min after CIP exposure, averaged from 40 tracked cells from a single representative biological replicate. (C) Average HU-mCherry fluorescence intensity along the cells’ long axis. (D) Kymograph heat map of relative HU-mCherry intensity distribution over time. (**E**) DNA compaction phenotype distribution for baseline unchallenged cells (dots) and cells after CIP exposure (lines). Dots and lines represent means from three biological replicates, while error bars and shaded regions indicate standard deviation. A.u., arbitrary unit.

To establish a timeline of chromosomal reorganization after CIP exposure, we initially used a widefield microscope coupled with a microfluidic setup that allowed direct administration of CIP (10 μg/ml) during image acquisition (see “Materials and methods” section). Prior to CIP exposure, most cells displayed two clearly separated nucleoids, one in each cell half, often with two distinct nucleoid lobes each (Fig. 1A, 0 min). Following CIP exposure, we observed extensive chromosomal reorganization, culminating in a dense nucleoid structure at midcell (Fig. 1A, 16 min). To quantify this reorganization, we analyzed average DNA distribution along the cells’ long axis throughout the timelapse and found that cells consistently reorganized their DNA within 8–20 min post-CIP exposure (Fig. 1B).

To improve focus stability and temporal resolution without notable photobleaching, we switched to a spinning disk microscope with an automatic focus system (see “Materials and methods” section). Using this setup, we imaged live cells at 2-min intervals starting 10 min post-CIP exposure. Improved focus stability led to better separation of DNA foci represented by HU-mCherry fluorescence, allowing for identification of distinct nucleoids and nucleoid lobes. This led us to discover that the dense nucleoid structure at midcell resulted from a highly organized, stepwise compaction process and not from a sudden event. In cells with four distinct DNA foci, the two chromosomal foci within each cell half initially fused and compacted at the quarter positions (Fig. 1C and D, 12–14 min). Subsequently, these two remaining DNA foci migrated towards midcell and fused (Fig. 1C and D, and Supplementary Video S1).

After discovering this stepwise reorganization, we utilized 15-s interval live-cell imaging to examine the temporal distribution of DNA compaction phenotypes. We defined four phenotypes: (i) multifocal distribution, common in unchallenged cells; (ii) quarter-position compaction, the first reorganization step; (iii) midcell compaction, the second reorganization step; and (iv) periseptal compaction, a midcell compaction variant where septum formation inhibits complete convergence at the cell center (Supplementary Fig. S1, also “Materials and methods” section). Prior to exposure, most wild-type cells displayed multifocal distribution (Fig. 1E). After 8 min of CIP exposure, many cells began transitioning to the quarter-position compaction phenotype. More than 20% of cells exhibited this phenotype between 10 and 18 min after CIP exposure. Most cells quickly transitioned from quarter-position to midcell compaction, while those with septum formation transitioned to periseptal compaction, marking the endpoints of the DNA reorganization. At 18 min after CIP exposure, the multifocal distribution phenotype had vanished, and the majority of cells exhibited midcell compaction. The midcell compaction was not reversible at this CIP dose (10 μg/ml), as it persisted for several hours following a 1-min pulse exposure (Supplementary Video S2). To highlight the persistent, dense midcell endpoint of this ordered cellular response, we have termed the response “DNA supercompaction.”

### DNA supercompaction progression rate depends on DNA damage severity across multiple genotoxic agents

DNA supercompaction, as we have just defined, initially appears visually distinct from the compaction responses demonstrated following exposure to other genotoxic agents such as UV [18], MMC [21] bleomycin [20], γ-irradiation [51], or I-SceI-induced damage [52]. We hypothesized that these perceived visual distinctions result from variations in experimental conditions—such as dosage and exposure time—and that they therefore reflect differences in progression of the DNA supercompaction response rather than inherent differences in cellular mechanisms. Consequently, we aimed to test whether DNA supercompaction progression rate is directly linked to DNA damage severity and thus to the concentration and potency of genotoxic agents.

**Figure 2.**
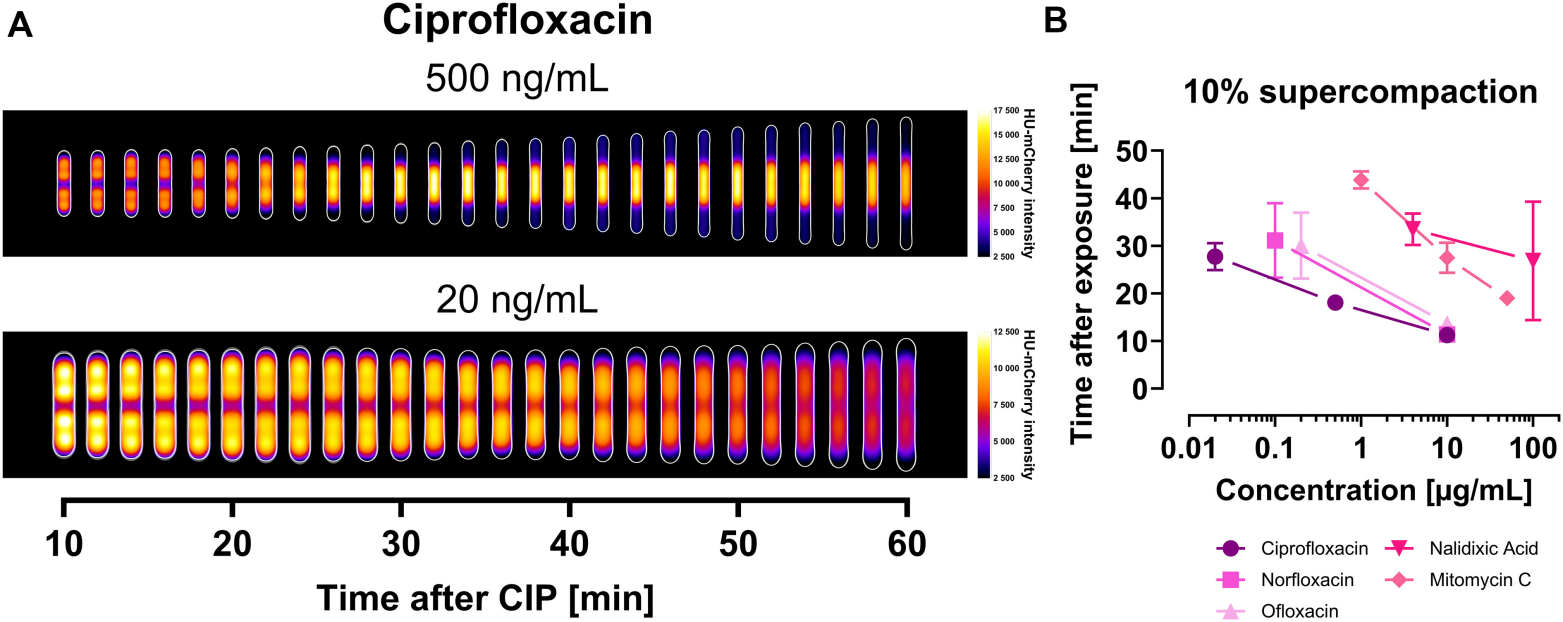
Progression rate of DNA supercompaction depends on the concentration of genotoxic agents. Wild-type cells (KV21) were grown in LB at 37°C and imaging started 10 min after exposure to the genotoxic agent. (**A**) Kymograph heat map of HU-mCherry intensity distribution for cells exposed to either an intermediate dose (500 ng/ml) of CIP or to the MIC dose (20 ng/ml). Results at different time points are averaged from 373 to 738 cells for the intermediate dose, and from 452 to 1164 cells for the MIC dose, in both cases from single representative biological replicates (see “Materials and methods” section). (**B**) Relationship between genotoxic agent concentration and DNA supercompaction progression rate, quantified as time required for 10% of cells to reach DNA supercompaction endpoints (midcell or periseptal compaction). Presented results are different concentrations of the genotoxic agents CIP, norfloxacin, ofloxacin, nalidixic acid, and MMC. Symbols represent means from 2 to 3 biological replicates; error bars indicate standard deviation.

Consistent with our hypothesis, we found that DNA supercompaction progression rate depends on the CIP concentration. A high dose (10 μg/ml) prompted most cells to reach supercompaction endpoints—exhibiting midcell or periseptal compaction—within 18 min of exposure (Fig. 1D and E), while intermediate (500 ng/ml) and lower (MIC, 20 ng/ml) concentrations extended the time window to ∼30 and 50 min, respectively (Fig. 2A).

Notably, we found that other genotoxic agents could also induce the DNA supercompaction response, as evident when using high concentrations 25–100 times the MIC (Supplementary Fig. S2). The fluoroquinolones norfloxacin and ofloxacin (10 μg/ml) induced rapid supercompaction comparable to CIP, while the less potent [53–55] quinolone nalidixic acid (100 μg/ml) and the DNA crosslinker [56] MMC (50 μg/ml) both prompted slower supercompaction progression. Importantly, all agents demonstrated concentration-dependent DNA supercompaction progression rates. To quantify this dependency, we measured the time required for 10% of cells to reach supercompaction endpoints (Fig. 2B)—a reliable metric for response initiation and progression rate. Less than 5% of unchallenged control cells exhibited these endpoints (Fig. 1E). Across all tested agents, higher concentrations consistently induced more rapid progression (Fig. 2B). Overall, we find that efficient DSB-inducers like CIP and other fluoroquinolones trigger rapid progression, while less effective DSB-inducers like nalidixic acid and MMC induce slower supercompaction responses. Our findings thus establish that DNA supercompaction progression rate depends on DNA damage severity.

### DNA supercompaction is dependent on RecN

We, and others, have previously demonstrated that reorganization of DNA following certain types of DNA damage largely depends on the SMC-like protein RecN [18–20]. Therefore, we sought to determine if RecN is essential for the DNA supercompaction observed after CIP exposure. We grew a *ΔrecN* strain expressing HU-mCherry (MR16 or KV68) to exponential phase and imaged the cells using both widefield and spinning disk fluorescence microscopy, following the same procedures as for the wild-type cells.

**Figure 3.**
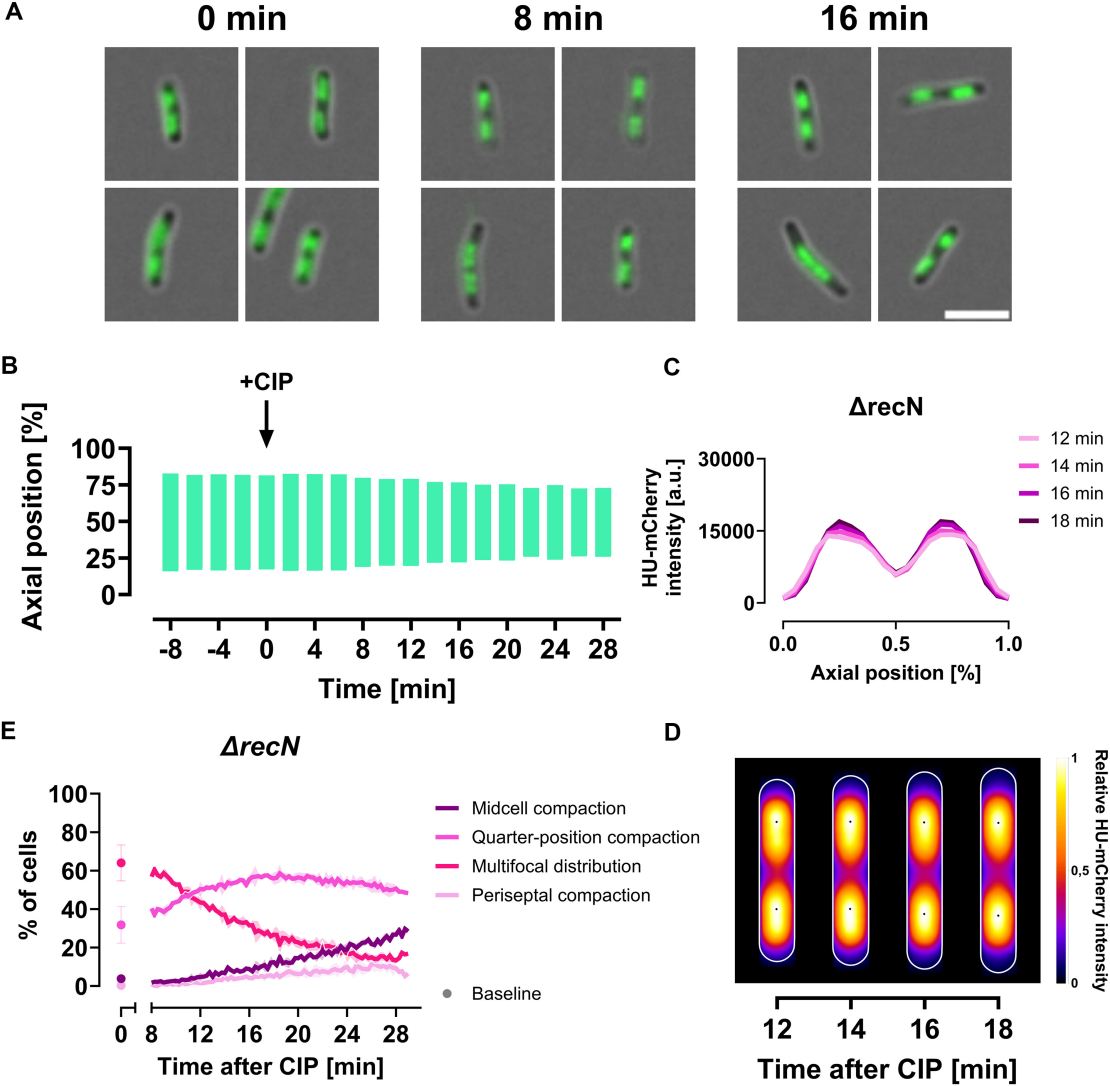
*ΔrecN* cells exhibit limited nucleoid reorganization after CIP exposure. All cells were grown in LB at 37°C and imaged at 2-minute intervals using live-cell imaging. (**A** and **B**) Cells (MR16) were immobilized in microfluidic channel slides and imaged using widefield microscopy. (A) Representative images of *ΔrecN* cells at 0, 8, and 16 min after CIP exposure, showing HU-mCherry fluorescence (green) to represent DNA distribution. Scale bar: 5 μm. (**B**) Analysis of DNA distribution along the cells’ long axis before and after CIP, quantified by measuring the distance between the outer bounds of symmetrical fluorescence peaks at 80% of maximum averaged intensity for each time point. Results are averaged from 17 to 61 cells from a single representative biological replicate (see “Materials and methods” section for detailed explanation). (**C** and **D**) Cells (KV68) immobilized on agar pads and imaged using spinning disk microscopy. Results are shown from images captured 12, 14, 16, and 18 min after CIP exposure, averaged from 40 tracked cells from a single representative biological replicate. (C) Average HU-mCherry fluorescence intensity along the cells’ long axis. (D) Kymograph heat map of relative HU-mCherry intensity distribution over time. (**E**) DNA compaction phenotype distribution for *ΔrecN* cells (KV68) at unchallenged baseline (dots) and after CIP exposure (lines). Dots and lines represent means from three biological replicates; error bars and shaded regions indicate standard deviation. A.u., arbitrary unit.

In striking contrast to wild-type cells, *ΔrecN* cells barely exhibited any midcell compaction after CIP exposure (Fig. 3A and B). Analyses of axial distribution and heat maps of HU-mCherry fluorescence from 40 tracked cells confirmed the non-responsive phenotype of *ΔrecN* cells (Fig. 3C and D). Quantifying the DNA compaction phenotypes, we found that *ΔrecN* cells only managed to initiate quarter-position compaction, but this occurred without the temporal control seen in the wild-type cells (Fig. 3E). Instead, the prevalence of this phenotype increased and persisted throughout the imaging period. Although a small fraction of *ΔrecN* cells was classified with the midcell compaction phenotype (Fig. 3E), these cells did not exhibit the same nucleoid density as wild-type cells with this phenotype, as evidenced by a wider distribution of DNA along the cells’ long axes (Supplementary Fig. S3A). Under unchallenged conditions, we did not observe any significant differences in the distribution of compaction phenotypes between wild-type and *ΔrecN* cells (Supplementary Fig. S3B). These findings led us to conclude that RecN is essential for the CIP-induced DNA supercompaction process.

We also aimed to assess the sensitivity of *ΔrecN* cells to CIP-induced DNA damage. As expected [26, 27, 57], we found *ΔrecN* cells (JW5416) to be extremely sensitive to short-term CIP exposure at our experimental dose (10 μg/ml), with survival rates plummeting to the detection limit within just 1 min (Supplementary Fig. S4A). Wild-type cells, which exhibit rapidly progressing DNA supercompaction at this dose, have greater tolerance to limited exposures to this CIP dose (Supplementary Fig. S4A). At a milder CIP dose (MIC, 20 ng/ml), much longer exposure times are required before the increased sensitivity of *ΔrecN* cells becomes prominent (Supplementary Fig. S4B). This timing corresponds with the slower progression of DNA supercompaction observed at this milder dose, though a causal relationship between these phenomena requires further investigation.

### RecN forms dynamic foci that migrate with the nucleoid towards midcell during DNA supercompaction

Previous studies have found that fluorescently tagged RecN typically forms foci at the cell poles or between nucleoids at midcell under unchallenged conditions [21, 33]. Following DNA damage by MMC, a majority of RecN foci were found to localize at the nucleoid [21, 33]. Given our discovery that RecN is essential for DNA supercompaction, we aimed to investigate its localization and dynamics after CIP exposure. To achieve this, we employed two low-copy number plasmids to express GFP-tagged RecN: pTF271 with an arabinose-inducible promoter (denoted pARA), and pSG101 with the native SOS-inducible RecN promoter (denoted pSOS). The pARA plasmid allowed us to study GFP–RecN dynamics in the earliest stages of the SOS response, while the pSOS plasmid ensured a native, timely expression of GFP–RecN throughout the SOS response [26]. We introduced these plasmids into the wild-type strain harboring chromosomally encoded HU-mCherry (KV21). Cells carrying the pARA plasmid (KV61) were exposed to arabinose (0.05%) for 60 min before CIP addition and live-cell spinning disk microscopy.

**Figure 4.**
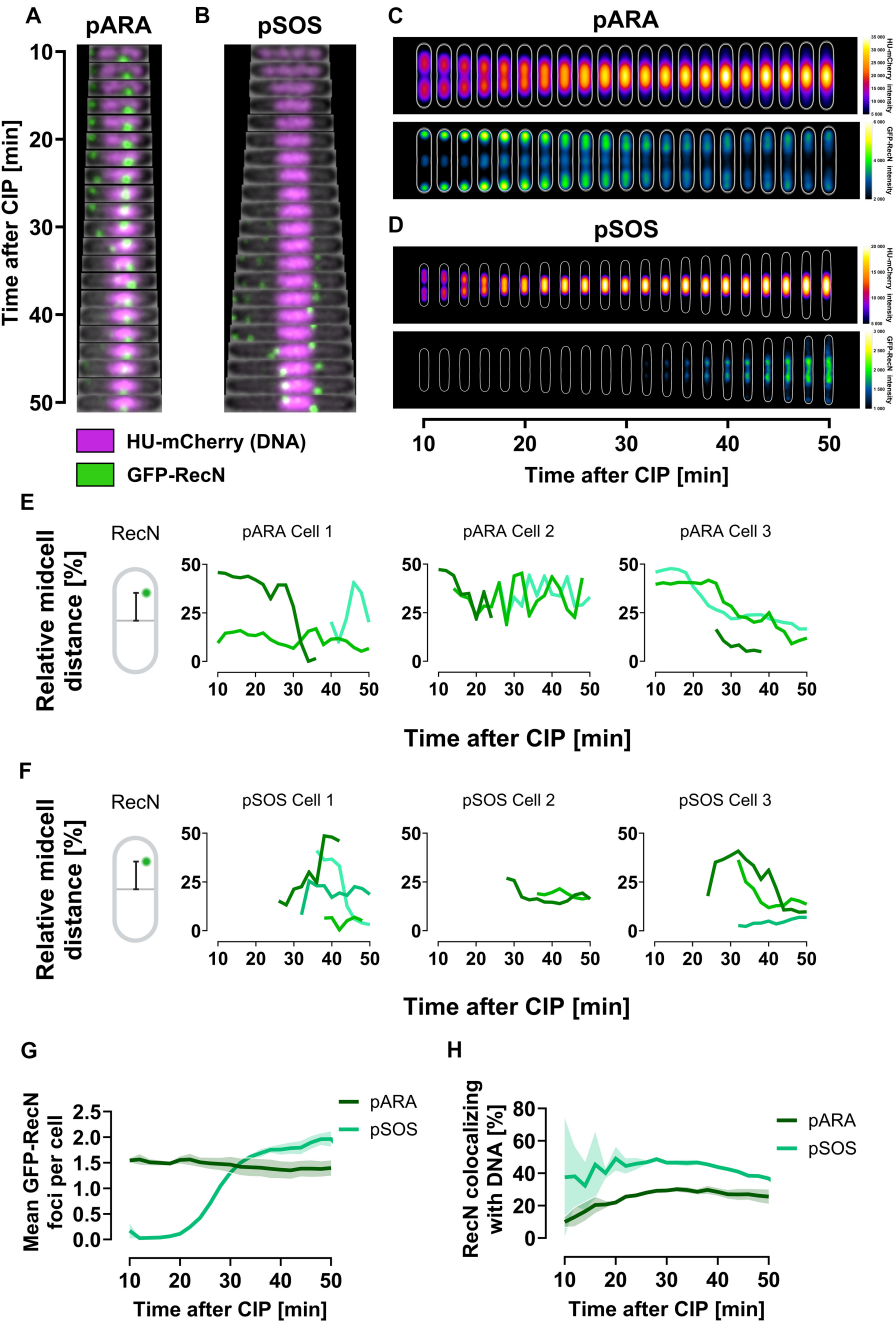
GFP–RecN foci exhibit dynamic movements in wild-type cells after CIP exposure. All cells were grown in LB at 37°C and imaged at 2-min intervals using live-cell spinning disk microscopy starting 10 min post-CIP exposure. Results represent three biological replicates. (**A** and **B**) Kymographs of representative cells showing GFP–RecN dynamics in relation to DNA organization when GFP–RecN is expressed from (A) pARA (KV61) or (B) pSOS (KV60). GFP–RecN fluorescence is shown in green, while DNA is represented by HU-mCherry fluorescence in magenta. (**C** and **D**) Kymograph heat map of HU-mCherry intensity distribution (upper panels) and GFP–RecN intensity distribution (lower panels) inside cells over time, when GFP-RecN is expressed from (C) pARA (KV61) and (D) pSOS (KV60). Results at different time points are averaged from 1092 to 1612 cells for the pARA strain (KV61), and from 328 to 480 cells for the pSOS strain (KV60), in both cases from single representative biological replicates (see “Materials and methods” section for detailed explanation). (**E** and **F**) GFP–RecN trajectories from representative cells showing the distance of GFP–RecN foci to midcell relative to cell length, when GFP–RecN is expressed from (E) pARA (KV61) and (F) pSOS (KV60). Each line represents a tracked focus. Midcell distance illustrations created in BioRender (https://BioRender.com/y04v494). (**G**) Mean number of GFP–RecN foci per cell versus time after CIP exposure for pARA and pSOS cells (KV61 and KV60). Lines represent means from three biological replicates, and shaded regions indicate SEM. (**H**) Percentage of GFP–RecN foci colocalizing with DNA versus time after CIP exposure for pARA and pSOS cells (KV61 and KV60). Data in the early time points for pSOS cells only represent a small number of cells with very weak foci presence, reflected by the large SEM.

As expected, under unchallenged conditions, GFP–RecN predominantly formed static foci at cell poles and occasionally at the center of dividing cells when expressed from pARA (Supplementary Fig. S5A and C). After CIP exposure, we observed a striking change: RecN foci at the poles migrated towards the nucleoids in a dynamic manner during DNA supercompaction (Fig. 4A and Supplementary Video S3). Analysis of fluorescence intensity from 328 to 480 cells revealed that GFP–RecN migration coincided with the onset of DNA supercompaction (Fig. 4C). Upon completion of supercompaction, the mean GFP–RecN fluorescence was evenly distributed between poles and the midcell nucleoid (Fig. 4C). Single-cell tracking showed that this wide distribution reflected the dynamic movement of RecN between poles and nucleoid occurring after DNA supercompaction was completed (Fig. 4E).

In wild-type cells carrying the pSOS plasmid (KV60), GFP–RecN expression begins at the onset of the SOS response. Consequently, GFP–RecN foci were not visible in these cells before or immediately after CIP exposure, contrasting cells with the pARA plasmid (Fig. 4A–D). This delay reflects the regulated induction of RecN expression in response to DNA damage. Importantly, the initial absence of visible foci does not indicate that GFP–RecN is inactive during the early stages after CIP exposure (see section “GFP–RecN is functional in DNA supercompaction”). As time progressed, the presence of GFP–RecN foci increased consistently across the population (Fig. 4G). After around 30 min, pSOS cells matched and later surpassed the mean number of GFP–RecN foci seen in pARA cells (Fig. 4G). These visible foci exhibited dynamic movements between cell poles and nucleoid (Fig. 4B and F, and Supplementary Video S4), consistent with observations in pARA cells.

Comparing heatmaps from pARA and pSOS cells (Fig. 4C and D), we found indications that the pSOS cells had a higher proportion of nucleoid-associated GFP–RecN foci than the pARA cells. Indeed, analysis of RecN and DNA colocalization revealed a consistently higher fraction of GFP–RecN foci localized at the nucleoid in the pSOS cells compared to the pARA cells (Fig. 4H). Additionally, pSOS cells exhibited faster DNA supercompaction than pARA cells. Although DNA supercompaction started 12–14 min after CIP exposure in both strains, the pARA cells took about twice as long as the pSOS cells to complete supercompaction (Fig. 4C and D, upper panels).

We also introduced both plasmids into a *ΔrecN* background expressing HU-mCherry and observed that GFP–RecN was able to drive DNA supercompaction in the absence of endogenous *recN*, albeit at a slower rate than in the wild-type background (Supplementary Fig. S6A D versus Fig. 4A–D). In the *ΔrecN* background, GFP–RecN foci were distributed more evenly across cells after supercompaction than in the wild-type background (Supplementary Fig. S6C and D, lower panels). The *ΔrecN* pSOS cells displayed fewer GFP–RecN foci per cell than wild-type pSOS cells (Supplementary Fig. S6G), accompanied by reduced colocalization with DNA (Supplementary Fig. S6H). In contrast, *ΔrecN* pARA cells maintained the same level of GFP–RecN foci as wild-type pARA cells (Supplementary Fig. S6G), while showing a slight increase in DNA colocalization (Supplementary Fig. S6H).

### GFP–RecN is functional in DNA supercompaction

The physiological relevance of GFP–RecN has been debated, with suggestions that foci at poles are aggregates not involved in biological activities [26]. We observed anomalies likely caused by GFP–RecN expression, including slower DNA supercompaction—especially in pARA cells (Fig. 4C)—which became more pronounced in a *ΔrecN* background (Supplementary Fig. S6C). However, GFP–RecN foci located at poles did respond to DNA damage and became highly dynamic after CIP exposure, even in a *ΔrecN* background (Fig. 4 and Supplementary Fig. S6). Importantly, GFP–RecN actively facilitates DNA supercompaction in the absence of native RecN, even before forming foci detectable under a microscope, as seen in *ΔrecN* pSOS cells (Supplementary Fig. S6B, D, and G). Differences in DNA supercompaction progression rates with and without native RecN may reflect suboptimal overall RecN levels or an imbalance between efficient native RecN and possibly a less efficient GFP-tagged version.

To survey native RecN and GFP–RecN levels in our strains, we performed western blotting. We compared protein levels in pARA- and pSOS-containing strains with their wild-type or *ΔrecN* background strains, both before and after 20 min of CIP exposure (Supplementary Fig. S7A and B). The pARA strains were incubated with arabinose (0.05%) for 60 min before CIP addition. Our results showed that overall RecN levels in pSOS and pARA strains were only modestly increased by ∼2–3 and 4 times, respectively, compared to the wild-type background without plasmid, after subtracting the signal from the *ΔrecN* negative control (Supplementary Fig. S7C). The *ΔrecN* strains carrying pSOS exhibited RecN levels comparable to that of the wild-type.

We next examined if GFP–RecN presence and increased overall RecN levels affect survival by performing spot assays using strains carrying either the pARA or pSOS plasmids and comparing them with their background strains. We found that these plasmids did not negatively affect survival rates in either wild-type or *ΔrecN* backgrounds after 20 min of CIP exposure (Supplementary Fig. S8). Additionally, GFP–RecN expression in *ΔrecN* backgrounds restored survival to wild-type levels. These results are consistent with previous findings in MMC-treated cells [21, 33].

Despite elevated overall RecN levels during DNA supercompaction in cells with pARA or pSOS plasmids, GFP–RecN still drives supercompaction, restores survival to wild-type levels, and exhibits dynamic movement between poles and supercompacted DNA. This indicates that GFP–RecN is functionally active in RecN-dependent processes, while potentially performing better when native RecN is present.

### RecA is essential for DNA supercompaction and RecN dynamics

Having established that GFP–RecN can drive DNA supercompaction, we next investigated the role of RecA in the dynamics of RecN. Previous studies showed that fluorescently tagged RecA, similar to GFP–RecN, typically localizes at cell poles under unchallenged conditions and migrates to the nucleoid after DNA damage [33]. In addition, RecN and RecA colocalize in nucleoid gaps after MMC treatment in *E. coli* [33], and RecA dynamics depend on RecN in *Caulobacter crescentus* and *Bacillus subtilis* [34, 58]. Based on these insights, we hypothesized that RecN dynamics after CIP exposure depend on RecA. To test this hypothesis, we performed live-cell imaging of GFP–RecN in a *ΔrecA* background harboring HU-mCherry.

**Figure 5.**
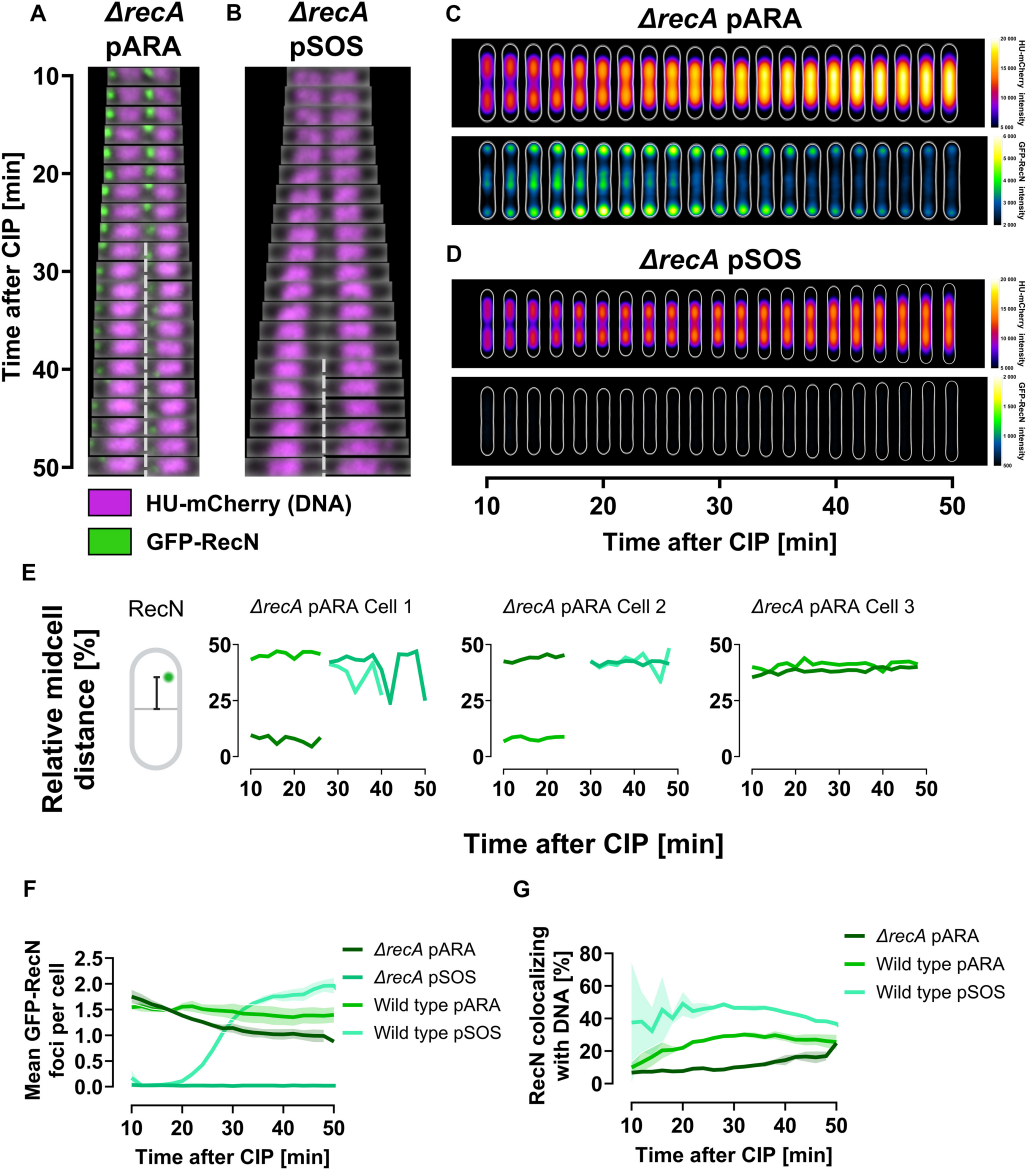
GFP–RecN foci dynamics after CIP exposure are diminished in *ΔrecA* cells. All cells were grown in LB at 37°C and imaged at 2-min intervals using live-cell spinning disk microscopy starting 10-min post-CIP exposure. Results represent three biological replicates. (**A** and **B**) Kymographs of representative *ΔrecA* cells showing GFP–RecN dynamics and DNA organization when GFP–RecN is expressed from (A) pARA (KV67) or (B) pSOS (KV66). GFP–RecN fluorescence is shown in green, while DNA is represented by HU-mCherry fluorescence in magenta. Dashed gray lines indicate cell division. (**C** and **D**) Kymograph heat map of HU-mCherry intensity distribution (upper panels) and GFP–RecN intensity distribution (lower panels) inside cells over time, when GFP–RecN is expressed from (C) pARA (KV67) and (D) pSOS (KV66). Results at different time points are averaged from 579 to 899 cells for the *ΔrecA* pARA strain (KV67), and from 513 to 894 cells for the *ΔrecA* pSOS strain (KV66), in both cases from single representative biological replicates (see “Materials and methods” section for detailed explanation). (**E**) GFP–RecN trajectories from representative *ΔrecA* pARA cells (KV67) showing the distance of GFP–RecN foci to midcell relative to cell length. Each line represent a tracked focus. Midcell distance illustration created in BioRender (https://BioRender.com/y04v494). (**F**) Mean number of GFP–RecN foci per cell versus time after CIP exposure for *ΔrecA* pARA and pSOS cells (KV67 and KV66). (**G**) Percentage of GFP–RecN foci colocalizing with DNA versus time after CIP exposure for *ΔrecA* pARA and pSOS cells (KV67 and KV66). Results for wild-type pARA and pSOS in (F) and (G) are the same as presented in Fig. 4. Lines represent means from three biological replicates, and shaded regions indicate SEM.

Strikingly, in the *ΔrecA* background, arabinose-induced GFP–RecN could not facilitate DNA supercompaction after CIP exposure (Fig. 5A and Supplementary Video S5). Although many cells displayed DNA at midcell after ∼25 min of exposure, it remained distributed across the cell length (Fig. 5C). This midcell localization was not due to DNA reorganization but was instead a consequence of continued cell division in the absence of SOS response induction (see Fig. 5A and B). Furthermore, GFP–RecN foci remained at cell poles or in nucleoid gaps after CIP exposure (Fig. 5A and C, and Supplementary Video S5). Single-cell analyses revealed diminished movement of GFP–RecN across the cell length in the *ΔrecA* background compared to the wild type (Fig. 5E versus Fig. 4E). As expected, *ΔrecA* cells harboring the pSOS plasmid did not produce any GFP–RecN foci because of the absence of SOS induction in this background (Fig. 5B and D). The findings above are consistent with observations in a *ΔrecN ΔrecA* background (Supplementary Fig. S9).

The mean number of arabinose-induced GFP–RecN foci remained largely unaffected by the absence of RecA during DNA supercompaction (Fig. 5F). However, the proportion of these foci colocalizing with DNA considerably decreased in the absence of RecA compared to wild-type or *ΔrecN* backgrounds (Fig. 5G), aligning with a previous report [21]. This reduced colocalization with DNA cannot be attributed to a deficiency in the formation of GFP–RecN foci.

In summary, the absence of RecA abolishes DNA supercompaction, even when GFP–RecN is artificially expressed from a plasmid. Additionally, it disrupts RecN localization and diminishes RecN dynamics. These findings indicate that RecN cannot function properly without either RecA, induction of the SOS response, or both.

### RecA has a multifaceted role in DNA supercompaction beyond SOS induction

Our investigation has established that RecN cannot function properly in DNA supercompaction without RecA. However, it remains unclear whether RecA’s role is limited to being an SOS response inducer or involves a direct interplay with RecN, as others have proposed [19, 21, 31, 33, 34, 58, 59]. To differentiate between these roles, we examined strains with temperature-regulated SOS induction that either possessed native RecA (*recA*^+^) or lacked RecA (*ΔrecA*) in a consistent genetic background. These strains featured two mutations in the *lexA* gene—*lexA41 (lexA3)*—which make LexA repressor cleavage, and thus SOS induction, depend solely on the temperature-sensitive activity of Lon protease rather than RecA [44].

**Figure 6.**
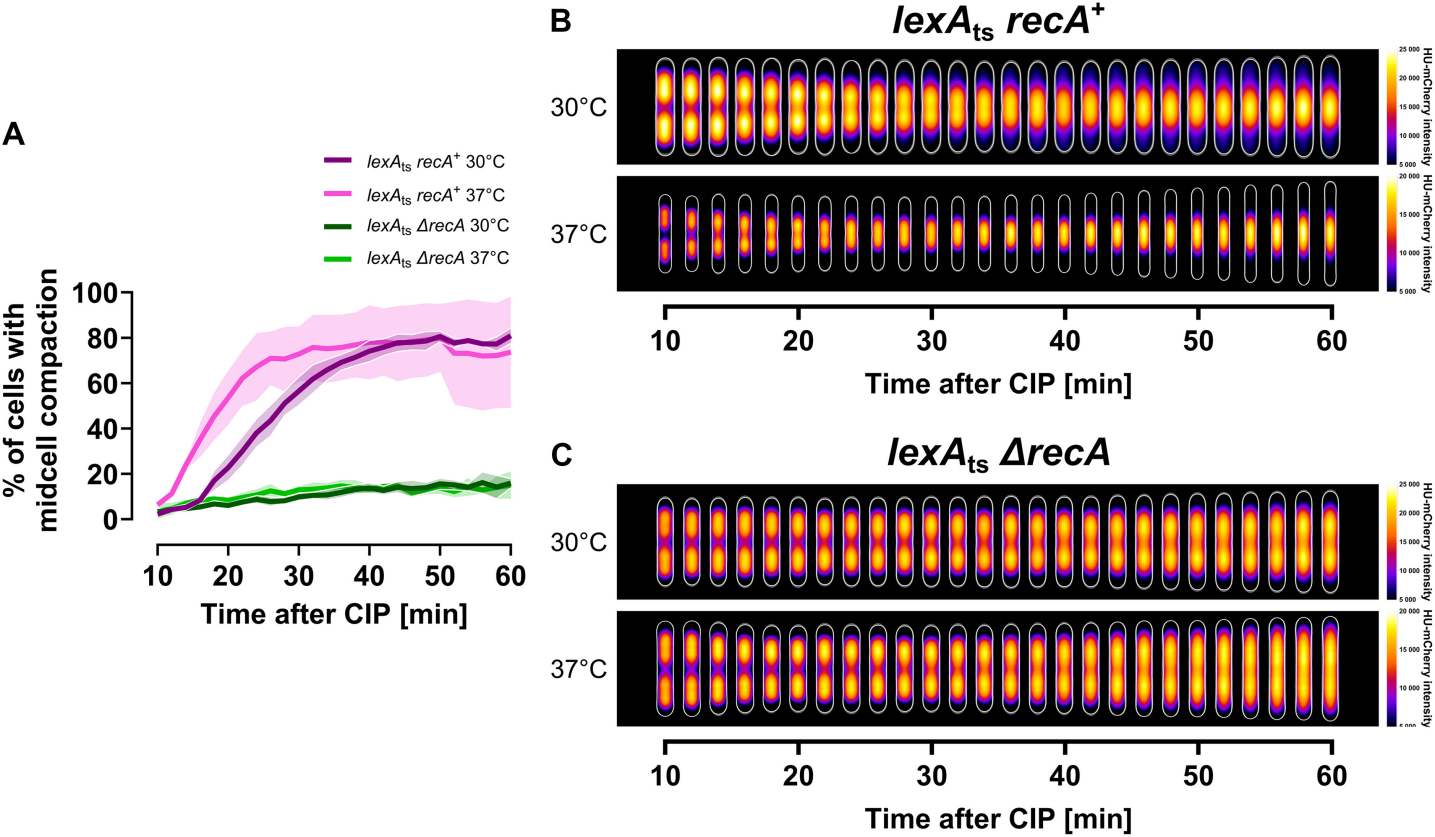
Effect of RecA on DNA supercompaction following CIP exposure in cells with temperature-regulated SOS induction (*lexA*_ts_). Comparison between strains that either possessed native RecA (*recA*^+^, KV90) or lacked RecA (*ΔrecA*, KV92). Cells were grown in LB at 30°C or 37°C and imaged at 2-min intervals using live-cell spinning disk microscopy starting 10-min post-CIP exposure, while being kept at the same temperature. (**A**) Quantification of cells displaying the midcell compaction phenotype after CIP exposure for each strain-temperature combination. Lines represent means from three biological replicates, while shaded regions indicate standard deviation. (**B** and **C**) Kymograph heat maps of HU-mCherry intensity distribution inside cells over time after CIP exposure at 30°C and 37°C for (B) *recA*^+^ cells (KV90) and (C) *ΔrecA* cells (KV92). Results at different time points are averaged from 127 to 928 cells for the *recA*^+^ strain (KV90), and from 145 to 328 cells for the *ΔrecA* strain (KV92), in both cases from single representative biological replicates (see “Materials and methods” section).

At an elevated temperature (37°C), the majority of *recA*^+^ cells (KV90) achieved midcell compaction within 20 min of CIP exposure (Fig. 6A and B). At a lower temperature (30°C), where Lon activity is low and the SOS response is presumably limited, *recA*^+^ cells required 30 min for the majority to exhibit midcell compaction (Fig. 6A and B). In stark contrast, *ΔrecA* cells (KV92) barely achieved any midcell compaction at either temperature within 60 min of CIP exposure (Fig. 6A and C). Whereas *recA*^+^ cells showed DNA supercompaction progression rates similar to the wild type (Supplementary Fig. S10A and B versus Fig. 1E), *ΔrecA* cells displayed abnormal distributions and lacked temporal control of compaction phenotypes, even though the SOS response was active (Supplementary Fig. S10C and D). These findings reveal that RecA plays a multifaceted role in the cellular DNA damage response, functioning not merely as an SOS response inducer but also as an integral component of the DNA supercompaction process itself.

### RecN and RecA interact and show frequent transient colocalization at nucleoid-associated positions during DNA supercompaction

Building on our observations of RecA’s essential role in DNA supercompaction, we aimed to elucidate the interplay between RecN and RecA. For this purpose, we performed live-cell spinning disk microscopy on cells expressing fluorescently tagged versions of both proteins. RecA-mCherry was expressed in tandem with the endogenous *recA* gene, while GFP–RecN was expressed from either the pARA or pSOS plasmid (KV79 and KV77). We confirmed that these strains exhibited DNA supercompaction after CIP exposure by imaging fixed cells stained with Hoechst 33258 (Supplementary Fig. S11).

**Figure 7.**
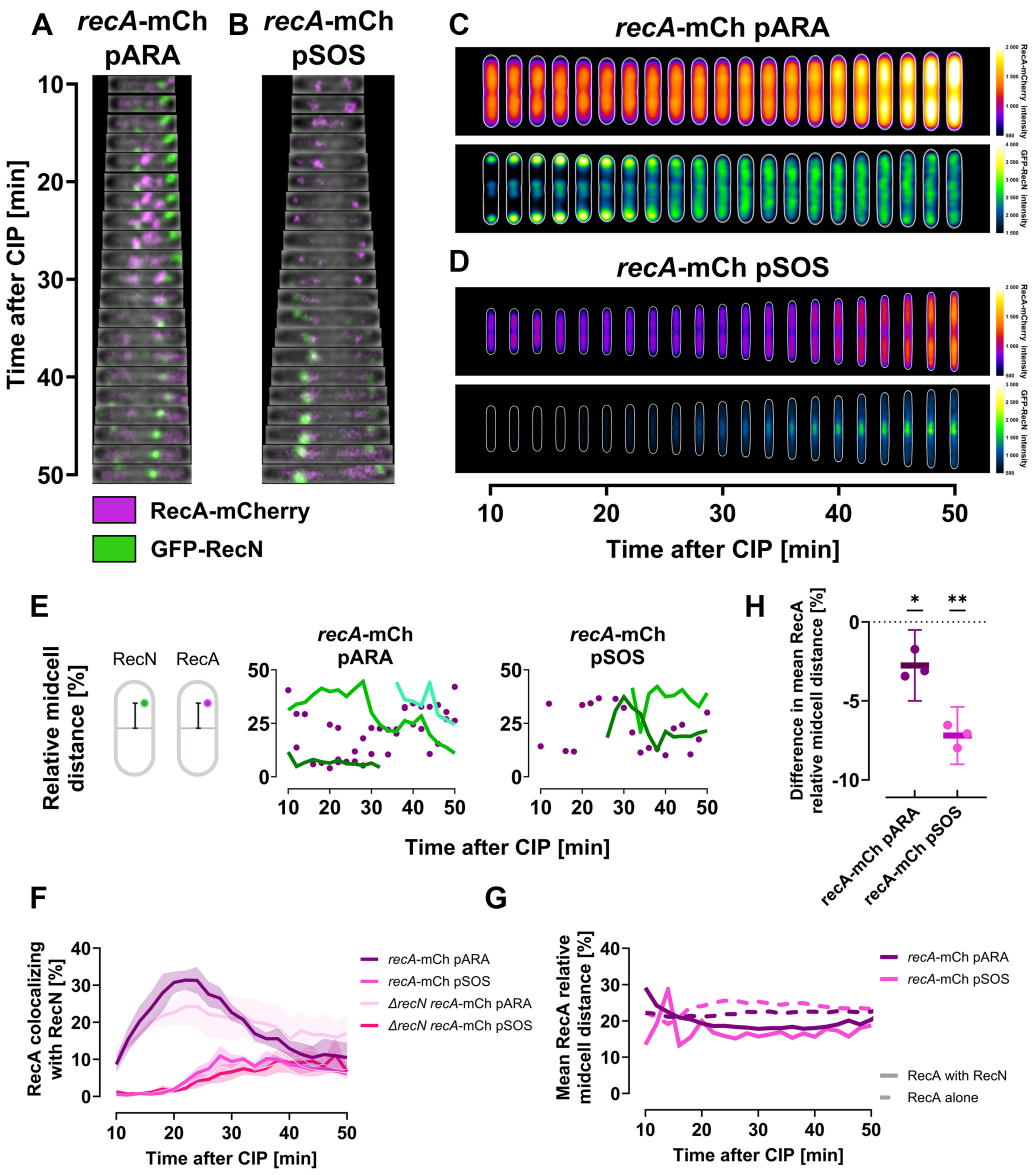
RecA-mCherry foci frequently and transiently colocalize with GFP–RecN foci after CIP exposure, with colocalizing RecA-mCherry foci positioned closer to midcell. All cells were grown in LB at 37°C, expressing RecA-mCherry chromosomally alongside native RecA. Cells were imaged at 2-min intervals starting 10 min post-CIP exposure using live-cell spinning disk microscopy. Results represent three biological replicates. (**A** and **B**) Kymographs of representative wild-type cells showing RecA-mCherry (magenta) and GFP–RecN (green) dynamics when GFP–RecN is expressed from either (A) pARA (KV79) or (B) pSOS (KV77). (**C** and **D**) Kymograph heat map of RecA-mCherry intensity distribution (upper panels) and GFP–RecN intensity distribution (lower panels) inside cells over time, when GFP–RecN is expressed from (C) pARA (KV79) or (D) pSOS (KV77). Results at different time points are averaged from 480 to 792 cells for the pARA strain (KV79), and from 231 to 398 cells for the pSOS strain (KV77), in both cases from single representative biological replicates (see “Materials and methods” section for detailed explanation). (**E**) GFP–RecN trajectories and RecA-mCherry localization for representative cells expressing GFP–RecN from pARA (KV79, left) and pSOS (KV77, right). Plots display the distance of foci to midcell relative to cell length, with GFP–RecN trajectories as green lines and RecA-mCherry foci as magenta dots. Midcell distance illustrations created in BioRender (https://BioRender.com/y04v494). (**F**) Percentage of RecA-mCherry foci colocalizing with GFP–RecN foci versus time after CIP exposure for wild-type and *ΔrecN* cells with pARA or pSOS plasmids. Lines represent means from three biological replicates, and shaded regions indicate SEM. (**G**) Mean distance of colocalizing (solid line) and noncolocalizing (dashed line) RecA-mCherry foci to midcell relative to cell length. Means are from three biological replicates. (**H**) Difference in relative midcell distance for colocalizing versus noncolocalizing RecA-mCherry foci. Negative values indicate that RecA-mCherry foci colocalizing with GFP–RecN are closer to midcell than noncolocalizing RecA-mCherry. Lines show means, dots represent replicates’ mean values, and error bars indicate 95% CIs; *P ≤ 0.05, **P ≤ 0.01.

RecA-mCherry foci displayed rapid kinetics, often appearing and disintegrating swiftly (Fig. 7A and B, and Supplementary Videos S6 and S7), which complicated tracking of individual foci to characterize their dynamics. These rapid kinetics are emphasized by the wide average distribution of RecA-mCherry across cells at all time points (Fig. 7C and D, upper panels), and the noticeable variation in the mean number of foci per cell (Supplementary Fig. S12A). Around 40 min after CIP exposure, single RecA-mCherry foci tended to disappear, while overall RecA-mCherry fluorescence increased and spread more evenly across the cells (Fig. 7C and D, upper panels). Consequently, we centered our analysis on the cellular localization of RecA-mCherry foci after CIP exposure and their potential colocalization with GFP–RecN foci.

We observed that RecA-mCherry frequently and transiently colocalized with GFP–RecN (Fig. 7E and F, Supplementary Videos S6 and S7, and Supplementary Fig. S13). In the pARA-carrying cells, we noted a remarkable spike in RecA-mCherry colocalizing with GFP–RecN, which coincided with the period of DNA supercompaction (Fig. 7F). The pSOS cells, with barely any detectable GFP–RecN foci during DNA supercompaction (Supplementary Fig. S12B), did not exhibit a similar colocalization spike. Nevertheless, after 40 min of CIP exposure, the pSOS cells achieved colocalization levels similar to the wild-type pARA cells (Fig. 7F).

Additionally, RecA-mCherry foci colocalizing with GFP–RecN were located significantly closer to midcell, and thus closer to the supercompacted DNA, than non-colocalizing RecA-mCherry foci in both pARA and pSOS cells (Fig. 7G and H). Colocalizing foci were 2.8% (95% CI: 0.5–5.0; *P*= 0.03) and 7.2% (95% CI: 5.4–9.0; *P*= 0.003) closer to midcell relative to cell length for wild-type pARA and pSOS cells, respectively. This proximity to midcell became particularly prominent after >20 min of CIP exposure, corresponding to the completion of DNA supercompaction (Fig. 7G and Supplementary Fig. S11). Lack of endogenous *recN* reduced but did not eliminate the midcell proximity differences in both pARA and pSOS cells (Supplementary Fig. S12C and D). Using a bacterial two-hybrid assay, we also demonstrated a strong direct interaction between RecN and RecA (Supplementary Fig. S14 and Supplementary Table S2), consistent with earlier studies [19, 30, 31]. In summary, these findings indicate that RecN and RecA interact and function cooperatively in proximity of DNA during supercompaction.

## Discussion

In this study, we demonstrate that exposure of *E. coli* cells to the fluoroquinolone antibiotic CIP leads to a major reorganization of DNA into a dense structure at midcell—a process we term DNA supercompaction. Live-cell fluorescence microscopy reveals that this process occurs in a highly organized manner consisting of two distinct steps: an initial quarter-position compaction followed by midcell compaction (Fig. 1). Given the uniformity of this process, we propose that DNA supercompaction is part of an active cellular response to severe DNA damage.

### DNA supercompaction is a universal response to severe DNA damage

DNA supercompaction progresses through an organized DNA rearrangement from multifocal distribution, via compaction at the quarter positions, to midcell compaction (see Fig. 1 and Supplementary Fig. S1). Our study demonstrates that DNA supercompaction is not exclusive to CIP exposure but constitutes a universal cellular response to severe DNA damage. We observed this response, which culminates in dense midcell compaction, also following exposure to other genotoxic agents, including norfloxacin, ofloxacin, nalidixic acid, and MMC (Supplementary Fig. S2). The rate of progression varied notably across the different agents (Fig. 2B). The fluoroquinolones—CIP, norfloxacin, and ofloxacin—are potent inducers of severe DNA damage through DSB formation [3]. At concentrations of 10 μg/ml they all prompted rapid supercompaction. In contrast, nalidixic acid—a less potent quinolone [53–55]—induced a much slower response at 100 μg/ml. MMC, damaging DNA through a different mechanism [56], triggered modest progression rates at 50 μg/ml. These variations highlight the relationship between genotoxic agent potency and supercompaction, where more potent agents accelerate progression.

If genotoxic agent potency dictates the progression rate of DNA supercompaction, then adjusting the agent concentration should alter the response. Our experiments confirm this idea, showing consistent acceleration of progression rates with increasing concentrations across the genotoxic agents tested (Fig. 2 and Supplementary Fig. S2). For example, most cells reached supercompaction endpoints in a concentration-dependent manner with CIP: 50 min at 20 ng/ml, 30 min at 500 ng/ml, and 18 min at 10 μg/ml (Figs 1D and E, and 2A). This concentration-dependent relationship aligns with recent findings by Thédié *et al.*, who demonstrated a correlation between DNA compaction and CIP concentrations at lower doses [60].

CIP has a well-known ability to induce DSBs by targeting topoisomerases, which are essential for managing supercoiling in the chromosome [2–3, 61]. In our experiments, exposure to CIP presumably generated numerous DSBs at rates influenced by CIP concentration. Previous research confirms that CIP induces DNA fragmentation—a consequence of DSB formation—in a concentration-dependent manner [62]. These observations, alongside our results (Figs 1 and 2), suggest that a greater number of DSBs, indicative of more severe DNA damage, accelerates the supercompaction response.

Less severe damage caused by mild UV exposure has been reported to only cause a transient and less uniform type of DNA compaction [18, 63]. Critically, inducing only a single DSB resulted in no visible nucleoid compaction [50]. Instead, the DSB-containing region and its homologous DNA were transported to midcell for repair [50]. These findings may imply that damaged DNA is universally reorganized for repair, with more severe damage leading to increased DNA transportation and a higher degree of compaction. Resolving midcell compaction might then necessitate repairing all damaged DNA—likely an overwhelming task amid rapid and extensive damage induction. Indeed, we found that supercompaction persisted for several hours (Supplementary Video S2) after a brief, high-dose CIP exposure (10 μg/ml).

### RecN is an essential facilitator of DNA supercompaction

We identify RecN as an essential facilitator of DNA supercompaction in response to severe DNA damage caused by CIP. Our high-resolution time-lapse imaging revealed that cells lacking RecN are unable to progress and complete the DNA supercompaction response, resulting in a lack of dense midcell compaction (Fig. 3A–D). Although a small fraction of *ΔrecN* cells exhibited a midcell compaction phenotype (Fig. 3E), their nucleoids were less dense than those of wild-type cells with this phenotype (Supplementary Fig. S3A). While *ΔrecN* cells could initiate a form of quarter-position compaction, they lacked the temporal control characteristic of wild-type cells for transitions between different compaction phenotypes (Fig. 3E). The occurrence of quarter-position compaction without RecN may indicate that other factors, such as nucleoid-associated proteins (NAPs), contribute to this process. Alternatively, the observed phenotype may arise from compensatory mechanisms activated in RecN’s absence, possibly diverging from the native DNA supercompaction process. Nonetheless, RecN is clearly necessary for the proper timing and completion of DNA supercompaction.

Previous studies have similarly highlighted a necessity for RecN in DNA compaction following exposure to various genotoxic agents, such as MMC [20], bleomycin [20], γ-irradiation [51], and UV-irradiation [18]. RecN is rapidly induced following DNA damage [13] and reaches high levels within 10 min of CIP exposure [24]. However, it undergoes rapid degradation by ClpXP protease, with a half-life of ∼10 min [26]. This swift yet transient presence at elevated levels indicates RecN’s vital role in the immediate DNA damage response.

To shed more light on the activity of RecN in the DNA supercompaction process, we tracked the localization of functionally active GFP-tagged RecN. We discovered that RecN foci migrated with the nucleoids towards midcell during DNA supercompaction and subsequently moved dynamically between poles and midcell (Fig. 4). RecN also frequently colocalized with the DNA (Fig. 4H), indicating activity on the DNA. Combined with our finding that cells lacking RecN cannot perform DNA supercompaction (Fig. 3), we suggest that RecN has an active role in reorganizing DNA after CIP exposure.

In vitro studies have shown that RecN can bind and bridge DNA molecules. Keyamura and Hishida demonstrated that purified *E. coli* RecN can bind both ssDNA and dsDNA, with a preference for ssDNA [30]. They proposed that RecN is recruited to ssDNA at DSBs, where it can topologically entrap a second dsDNA in an ATP-dependent manner [30]. Similar properties have been observed *in vitro* for *Deinococcus radiodurans* RecN, which can bridge dsDNA molecules through ATP-dependent activity [23, 32]. While the relevance of these *in vitro* findings for *in vivo* functions remains to be fully elucidated, our results, supported by other studies [19, 33], show that RecN requires the presence of DSBs to perform DNA compaction (Supplementary Fig. S5). This indicates that RecN’s activity in DNA supercompaction may be linked to the immediate DNA damage response. The extreme sensitivity of *ΔrecN* cells to short-term CIP exposure at a high dose (10 μg/ml) (Supplementary Fig. S4) further supports the notion that RecN’s role in supercompaction is vital to the repair of DSBs.

### RecA is essential for DNA supercompaction beyond SOS induction

RecA is another key actor in the bacterial response to DNA damage [5], and we have found that it is indispensable for DNA supercompaction (Fig. 5). One might expect RecA’s essentiality in this process to stem solely from its role as an inducer of the SOS response, which is critical for the expression of RecN and other repair proteins following DNA damage [12–14]. To distinguish RecA’s SOS inducing role from other functions, we used strains with temperature-sensitive SOS induction, where Lon protease, rather than RecA, regulates LexA inhibition [44]. In our experiments, *recA*^+^ cells successfully induced DNA supercompaction after CIP exposure, with most cells achieving midcell compaction within 20–30 min, depending on the temperature (Fig. 6A and B). On the other hand, barely any *ΔrecA* cells achieved midcell compaction within 60 min (Fig. 6A and C). Instead, these cells displayed abnormal distribution of compaction phenotypes and lacked the temporal control seen in wild-type and *recA*^+^ cells (Supplementary Fig. S10). Furthermore, arabinose-induced GFP–RecN expression failed to drive DNA supercompaction in a *ΔrecA* background (Fig. 5A and C), unlike in *ΔrecN* cells, signifying RecA’ role beyond SOS induction. While RecA does influence DNA supercompaction by inducing RecN expression through the SOS response, our findings reveal that RecA also plays an essential role in the induction and progression of DNA supercompaction through other mechanisms.

### Interplay between RecN and RecA in DNA supercompaction and repair

We observed frequent colocalization of RecN and RecA at nucleoid-associated positions following CIP exposure (Fig. 7) and found evidence indicating a direct interaction (Supplementary Fig. S14 and Supplementary Table S2). While RecA is known for its essential role in DSB repair through homology search and strand invasion [10, 64], the specifics of RecN’s involvement in this pathway are still being clarified [27, 28].

Recent studies on the interplay between RecN and RecA following DNA damage have predominantly centered on the hypothesis that RecN ensures close proximity between damaged and intact homologous DNA, thereby facilitating RecA’s homology search and strand invasion [19, 21, 31, 33, 34, 58, 59]. Efficient homology search likely requires both short-distance sampling through 1D sliding and hopping, and long-distance sampling through 3D intersegmental transfer on the DNA, depending on the location of the break(s) relative to intact homologous DNA [64, 65].

Vickridge *et al.* showed that RecN facilitates interaction between newly replicated strands of homologous DNA when DSBs arise at the replication fork, in a manner dependent on RecA [19]. In this case, the “zippering” activity of RecN could enable a rapid and effective homology search by RecA in the immediate vicinity of the DSB. In cases where DSBs are generated on already replicated and segregated DNA, RecA filaments appear to extend from the DSB throughout the cell, likely in the search for homologous DNA, as demonstrated in both *E. coli* and *C. crescentus* [34, 50, 52]. It has been indicated that the highly dynamic behavior of RecA filaments depends on the ATPase activity of RecN [34], and that this activity of RecN is also necessary for successful RecA-mediated repair [21, 33]. Moreover, Meddows *et al.* found that RecN is more vital for cell survival after the induction of three well-separated DSBs than after a single DSB [27]. In contrast, cells lacking RecA showed equal sensitivity to both one and three DSBs [27], indicating a more prominent role of RecN when multiple DSBs need repair. In our situation with numerous DSBs, a presumably large number of RecN proteins binding and bridging DNA in an attempt to repair the severe damage, could lead to the observed DNA supercompaction phenotype [66].

In vitro studies with purified *D. radiodurans* RecN support the *in vivo* studies and highlight the co-dependency between RecN and RecA. It was found that RecA’s function in DSB repair is dependent on the ATPase activity of RecN, and that ssDNA-bound RecA filaments heavily stimulate the ATPase activity of RecN [31]. It was suggested that this increased level of ATPase activity could power the movement of the RecN–RecA–ssDNA complex in the search for homologous DNA at distant sites [31]. Additionally, the confined, dense nucleoid may in itself increase efficiency of homology search between distant sites of the DNA [64, 67].

Although we cannot conclude mechanistic details concerning the RecA–RecN interaction from our study, it is evident that RecN and RecA exhibit a co-dependency not only in DSB repair but also in DNA supercompaction. The increased RecN–RecA colocalization we observed during DNA supercompaction (Fig. 7F) and the close proximity of colocalized RecA–RecN foci to midcell (Fig. 7G and H) may indicate cooperation at or near the compacted DNA.

### Functional and structural parallels between RecN and eukaryotic SMC proteins in compaction and repair

As an SMC-like protein, RecN belongs to a group of conserved proteins that are considered the primary drivers of DNA organization and compaction [68, 69]. Eukaryotic cells use multiple SMC complexes for tight chromosome organization, including the SMC5/6 complex [70]. Recent reports on SMC5/6 reveal roles and activities that are strikingly similar to those discussed for RecN. The SMC5/6 complex facilitates DSB repair, stabilizes arrested replication forks, and has a high affinity for ssDNA–dsDNA junctions and other recombination intermediates [71–73]. The complex can slide along the DNA and dimerize at junctions relevant for homologous recombination, and is then thought to compact damaged DNA regions by loop extrusion in an ATP-dependent manner (see [70] for review). Given the functional and structural resemblances between RecN and SMC5/6, it may be that they operate through similar mechanisms.

In conclusion, our study reveals that RecN, in concert with RecA, plays an essential role in the highly organized DNA supercompaction following severe DNA damage caused by CIP in *E. coli*. We propose that DNA compaction is part of a universal response to DSBs, with its degree dependent on the severity of the damage. Although a role of RecN in aiding RecA homology search could explain this effect, further investigation is needed. Given the rising prevalence of antibiotic resistance, particularly to fluoroquinolones, insights into bacterial survival responses to DNA-damaging antibiotics are crucial for developing preventive measures and novel treatments to combat resistance.

## Supplementary Material

gkaf437_Supplemental_Files

## Data Availability

All raw images, processed images, and corresponding analysis files underlying this article are available in the BioImage Archive [74] under accession number S-BIAD1780 at https://doi.org/10.6019/S-BIAD1780. Other data underlying this article will be shared on reasonable request to the corresponding author. All scripts and templates used for processing and analysis of images in this study are available in the Zenodo repository at https://doi.org/10.5281/zenodo.14063054.

## Supplementary data

Supplementary data are available at NAR online.
